# Type 2 Diabetes Self-Management Interventions Among Asian Americans in the United States: A Scoping Review

**DOI:** 10.1089/heq.2021.0083

**Published:** 2022-09-23

**Authors:** Dante Anthony Tolentino, Samreen Ali, Seo Young Jang, Celeste Kettaneh, Judith E. Smith

**Affiliations:** ^1^ National Clinician Scholars Program, University of Michigan, Ann Arbor, Michigan, USA.; ^2^ School of Nursing, University of Michigan, Ann Arbor, Michigan, USA.; ^3^ Institute for Healthcare Policy and Innovation, University of Michigan, Ann Arbor, Michigan, USA.; ^4^ Undergraduate Research Opportunity Program, University of Michigan, Ann Arbor, Michigan, USA.; ^5^ Taubman Health Sciences Library, University of Michigan, Ann Arbor, Michigan, USA.

**Keywords:** disaggregation, self-management, minority health, diabetes, self-care

## Abstract

**Introduction:**

Type 2 diabetes (T2D) is one of the leading causes of death among Asian Americans. Despite being a culturally diverse racial group with differences in history, language, religion, and values, Asian Americans are often viewed as a monolith. With the high prevalence rate of T2D, a careful examination of self-management interventions across Asian Americans is needed to develop effective and culturally sensitive interventions.

**Objective:**

To describe existing literature by examining study characteristics, different intervention components, and outcome measures of various T2D interventions among Asian Americans.

**Methods:**

Using Arksey and O'Malley's framework to ground this review, six online databases were used to identify studies.

**Results:**

A total of 18 publications were included. Thirteen studies were published after 2013, with 44% and 22% of these studies focused on Chinese Americans and Korean Americans. We found a lack of geographic diversity in the location of the studies. Majority of the participants were females. Most of the interventions were implemented in person. Licensed health care providers were the most common interventionists, with a number of studies using community health workers. Outcome measures focused on three key areas: physiological, psychosocial and behavioral, and program-related outcomes. Many of the studies measured changes in HbA1C, self-efficacy, distress, depression, and quality of life. Overall, we saw improvements in physiological measures in most of the studies. For example, majority of the studies showed a decline in the participants' HbA1C. Most studies showed an increase or improvement in healthy behaviors. Studies that measured efficacy, knowledge, attitude, motivation, quality of life, or general health showed improvement from baseline. All the studies that measured distress or depression showed a reduction of symptoms postintervention.

**Conclusion:**

Overall, we found that culturally tailored interventions that focus on specific Asian American subpopulations saw an improvement in physiological, psychosocial, or behavioral measures. There were several gaps in the existing T2D self-management programs or interventions among Asian Americans studied in the United States. Based on our analysis, we recommend when designing or implementing self-management interventions among Asian Americans, considerations should be made for targeted recruitment for understudied Asian American subgroups, gender, and location.

## Introduction

Type 2 diabetes (T2D) affects 11.4% of Asian Americans; it is the fifth leading cause of death in this group.^1^ Asian Americans living with T2D face multiple challenges, including language barriers, literacy and numeracy issues, access to care, cultural attitudes and beliefs, technology access, alternative health pathways, and dietary issues.^2–4^ Despite the increasing prevalence of T2D, meaningful differences in T2D challenges and needs are often masked among Asian Americans. Under recognition of their health care needs is caused by an oversimplification of their diversity; they are treated as a homogenous group even if they are socially, culturally, and economically diverse, representing more than 30 ethnic groups from over 20 countries.^5^ For instance, a closer look at the age-sex adjusted diabetes prevalence across Asian Americans show heterogeneity: 14.0% for East Asians, 22.4% for Southeast Asians, and 23.3% for South Asians.^1,6^

Living with T2D requires multifaceted decision-making, lifestyle modification, and behavior changes. Diabetes Self-Management Programs (DSMPs) offer the foundation to acquire knowledge and skills for self-care. They aim to help individuals manage their disease by providing them the opportunity to develop self-care behaviors and coping skills.^7,8^

Implementing DSMPs has significantly improved self-care behaviors by fostering healthy behaviors, increasing physical activity, increasing use of primary care and preventive services, and enhancing self-efficacy.^8^ But most of the DSMPs have not explicitly been tailored to Asian-Americans' unique and diverse needs with T2D. Given the diverse cultures and the number of Asian Americans impacted by T2D, an explicit characterization and understanding of DSMPs are needed; doing so may provide effective and culturally sensitive interventions to mitigate the complications from T2D. However, we know of no study that has synthesized the availability and impact of self-management interventions on Asian Americans.

Thus, the purpose of our study was to conduct a scoping review to systematically examine diabetes self-management interventions and outcomes targeted at Asian Americans and identify any existing gaps in knowledge. Specifically, we described existing literature by examining study characteristics and identifying the different components and outcome measures of the various T2D programs or interventions among Asian Americans.

## Methods

### Protocol and registration

We conducted a scoping review because self-management among Asian Americans is broad and heterogeneous in nature. We followed the framework established by Arksey and O'Malley^9^ with the following steps: (1) formulated the research question; (2) identified relevant studies; (3) selected the studies; (4) charted the data; and (5) collated, summarized, and reported of the results. We did not consult an expert, an optional step in Arksey and O'Malley's framework in this scoping review protocol. We published the final protocol with the Open Science Framework, and it can be accessed through this link: https://osf.io/c2ras^10^

### Eligibility criteria

The review was conducted following the PRISMA-ScR (Preferred Reporting Items for Systematic reviews and Meta-Analyses extension for Scoping Reviews) checklist and explanation^11^ and the updated PRISMA 2020 for the flow diagram.^12^ We used a two-stage screening process to assess the relevance of publications identified in the search.

We included studies on Asian Americans with T2D and focused on self- or family management dimensions. Specifically, we included peer-reviewed journal articles that were (1) primary studies of any research design (quantitative, qualitative, and mixed-methods), (2) written in English, (3) involved adult participants (≥18 years old and older), and (4) described or implemented a self- or family-management intervention for Asian Americans with T2D. No publication date limitations were included. We excluded other reviews (systematic or other literature reviews), commentary, opinion articles without original results, letters, conference abstracts, position papers/position statements, book/book chapters, and non-English articles.

### Information sources and search

We queried six databases on December 7, 2020: Medline (OVID; Wolters Kluwer, New York, NY); CINAHL Complete (EBSCOhost; EBSCO, Ipswich, MA); PsycInfo (EBSCOhost; EBSCO); Cochrane Library (Wiley, Hoboken, NJ); Scopus (Scopus.com; Elsevier, New York, NY); and Embase (embase.com; Elsevier). No date or language filters were applied. The search strategies were drafted by an informationist (J.E.S.) and further refined through team discussion. The primary search was conducted in Medline and combined controlled vocabulary (MeSH terms) with keywords in the title, abstract, and author-supplied keywords. Sample keywords included exercise and lifestyle. Examples of MeSH terms included “self-management,” and “self-care,” and “health behavior.” Searches in the additional databases were translations of the Medline search.

All search strategies are available in Supplementary Appendix SA1 and were rerun on June 17–18, 2021. We used the same search method, except that we narrowed the searches to 2021 onward. Citations were exported to Endnote X9 (Clarivate Analytics) and subsequently deduplicated. Citations were then imported into Distiller SR (Evidence Partners, Ottawa, Canada), an internet-based software, for screening.

### Selection of sources of evidence

#### Title and abstract screening

For the first level screening, we reviewed the title and abstract of the citations. Using DistillerSR, we developed a screening form. Two team members (D.A.T. and S.Y.J.) calibrated and pilot-tested the form using 10 random citations, reaching an inter-rater reliability score of *kappa*=1 after resolving inconsistencies in the form. Four reviewers (D.A.T., S.Y.J., S.A., C.K.) independently screened the title and abstract of each citation. Two reviewers needed to independently agree to exclude articles and advance articles to full-text screening. Disputes or disagreements were resolved by discussion with other screeners until a consensus was reached.

#### Full-text screening and eligibility

After the title and abstract screening, all relevant citations were screened for subsequent full-text review (level 2 screening). We also developed the screening form in DistillerSR to confirm relevance. Two team members (D.A.T. and S.A.) calibrated the full-text screening form until reaching inter-rater reliability of *kappa*=0.80. Similar to the title and abstract screening form, 10 random citations were used to calibrate and resolve any inconsistencies in the form. The same four reviewers (D.A.T., S.Y.J., S.A., C.K.) independently screened the full-text articles, and any disputes were resolved by consensus with all the scoping review members.

### Data charting process

The first author (D.A.T.) developed a standard data-charting form to determine which variables to extract. The form captured the relevant information on key study characteristics and detailed information describing the self-management interventions, programs, and outcomes for each included article.

The first author plus one other reviewer (S.Y.J., S.A., or C.K.) independently charted data from each eligible article. Any disagreements were resolved through discussion among all the scoping review members. Data charting was implemented using Google Sheet, a cloud-based spreadsheet system that allows multiple people to edit the same document simultaneously.

### Data items

The data-charting form captured relevant information on key study characteristics. We abstracted data on article characteristics (e.g., year of publication, context/background, aim, conceptual framework, and method), demographic characteristics (e.g., setting/geography, sample size, mean age, gender, education, income, Asian American subpopulation), intervention typology (e.g., intervention type, intervention delivery, interventionist, intervention components, significance), outcome typology (e.g., outcome measures, type of measurement used, change of outcome from baseline, significant findings, and measurement timeline), and other key results.

### Synthesis of results

To synthesize results, studies were grouped by different dimensions: (1) by study characteristics; (2) by aims, framework, study design; (3) intervention components and types; (4) outcome measures.

To synthesize the intervention components, we used the Diabetes Self-Management Education and Support (DSME/S) algorithm steps from the Joint Position Statement^13^ and American Diabetes Association (ADA) DSME/S Standards^14^ as a framework to categorize each of the studies' intervention components. We used 11 different themes to classify the intervention components: (A) Assessment and education (including assessment of cultural beliefs, health beliefs, current knowledge, physical limitations, family support, financial status, medical history, literacy, numeracy); (B) Medications; (C) Monitoring of blood glucose; (D) Physical activity; (E) Preventing, detecting and treating acute and chronic complications; (F) Nutrition; (G) Risk reduction (e.g., smoking cessation, foot care); (H) Developing personal strategies to address psychosocial issues and concerns; (I) Developing personal strategies to promote health and behavior change; (J) Review and reinforce treatment goals and self-management needs; and (K) Others.

We presented results in narrative format, tables, and visual representation. The following section presents our results as descriptive analyses with a complete list of included studies and summaries.

## Results

### Selection of sources of evidence

**FIG. 1.**
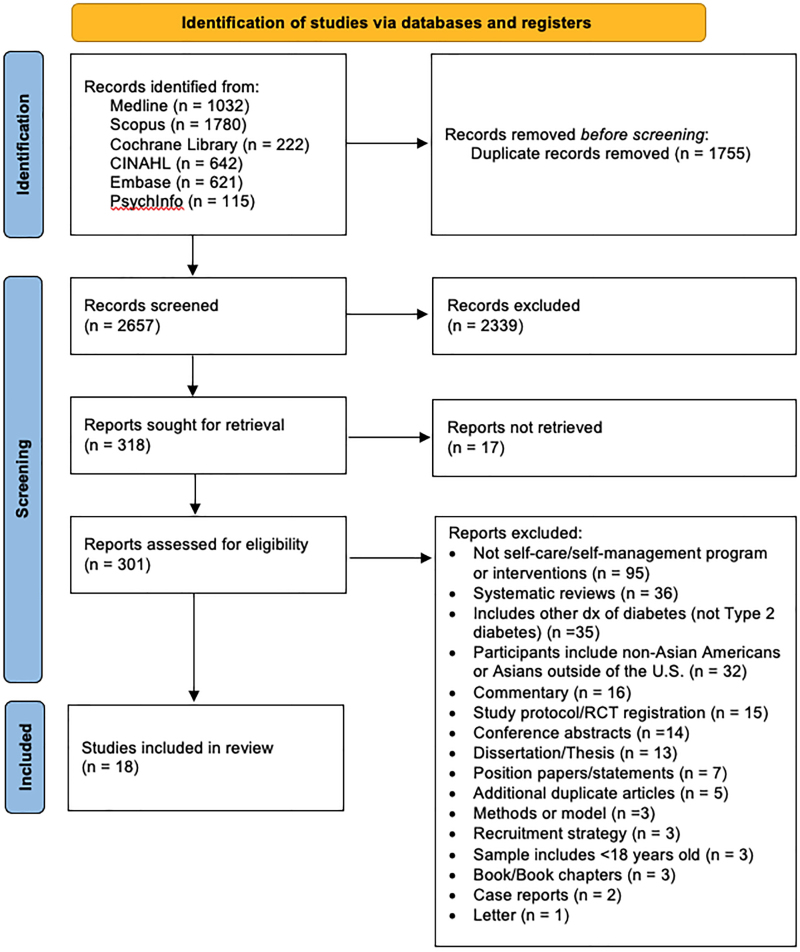
PRISMA 2020 flow diagram. PRISMA, Preferred Reporting Items for Systematic reviews and Meta-Analyses.

We found 4412 records in database searching. After removing duplicates, we screened 2657 records, from which we reviewed 301 full-text documents, and finally included 18 articles that were considered eligible for this review (Fig. 1).^15–31^

We excluded 283 studies from our review, and we listed reasons for exclusion in Figure 1. We excluded studies because they did not contain T2D self-management interventions or programs; some were types of systematic or literature reviews, studies included other types of diabetes other than T2D, non-Asian American participants, and others. We excluded additional five studies because they were duplicate articles that were not captured by the initial duplicate screening.

### Characteristics of sources of evidence

The different T2D self-management interventions implemented among Asian-Americans in the United States are described in detail in Tables 1–5 with the year published, aims, the framework used, setting, intervention participants demographic information, intervention components, and outcome measures.

**Table 1.** Demographic Information

Author	Asian American group representation	Geographic setting	Gender representation	Mean age of participants (years)	Most frequent education attainment of participants	Median income level	Primary language spoken^a^	Sample size
Bender et al. (2017)	Filipino American	San Francisco, CA	63% Female	57.4	78% with college education	Not reported	English	45
Chesla et al. (2014)	Chinese American	San Francisco, CA	56% Female	61.0	12th Grade level	87% Household income less than $50,000 annually	Cantonese	178
Chesla et al. (2013)	Chinese American	San Francisco, CA	59% Female	64.5	12 Years (mean)	88% Income less than $50,000 annually	Cantonese	145
Culhane-Pera et al. (2005)	Hmong American	Minnesota	100% Female	58.6	Not reported	Not reported	Hmong	277
Ho et al. (2021)	Chinese American	San Francisco, CA	67% Female	53% (61–70 years old)	40% Less than high school	Not reported	Cantonese	15
Ho et al. (2020)	Chinese American	San Francisco, CA	73% Female	61.1	64% High school	Income less than $30,000 annually	Cantonese	18
Inouye et al. (2015)	Asian American Pacific Islander	Hawaii	56.7% Female	57.0	33.3% College education	Not reported	Not reported	207
Islam et al. (2013)	Bangladeshi American	New York	57.7% Female	53.4	38.5% Graduate degree and beyond	Income less than $25,000 (although more than 50% not reported)	Bengali	26
Ivey et al. (2012)	Chinese American	Oakland, CA	60.9% Female	66.5	65.2% Less than high school	Not reported	Cantonese, Mandarin, or English	92
Kim et al. (2016)	Korean American	Baltimore, MD	59.1% Male	59.1	13.5 Years (mean)	Income $3807.00 monthly	Korean	250
Kim et al. (2015)	Korean American	Baltimore, MD	59.1% Male	59.1	13.5 Years (mean)	Income $3807.00 monthly	Korean	209
Kim et al. (2009)	Korean American	Baltimore-Washington, MD	44.3% Female	56.4	48.1% with college education or	Income less than $40,000 annually	Korean	79
Kwan et al. (2014)	Chinese American	San Francisco, CA	56.4% Female	60.8	12.25 Years (mean)	88% Less than $50,000	Cantonese	163
Le et al. (2013)	Chinese American (majority—77%)	Boston, MA	54.1% Female	62.8	36.1% Less than high school	Income $56,336.40	Multiple languages	327
Song et al. (2010)	Korean American	Baltimore-Washington, MD	44.3% Female	56. 4	Not reported	Not reported	Korean or English	79
Tomioka et al. (2014)	Filipino American (majority—92%)	Hawaii	87% Female	73.0	56.3% Less than high school	Not reported	Multiple languages	96
Wang and Chan (2005)	Chinese American	Hawaii	51.5% Female	68.8	57.6% High school graduate and beyond	81.8% Household income less than $1001 monthly	Cantonese, Mandarin, or Taiwanese	40
Yomogida et al. (2015)	Asian Americans	Hawaii	56.7% Female	41.58%: 60–69 Years old	33% College graduate	Not reported	Not reported	207

^a^ Data for primary language spoken were taken from demographic characteristics (if reported) or in the Methods section (e.g., inclusion criteria, intervention, etc.) of the study.

### Results of individual sources of evidence

#### Study characteristics

Eighteen studies included self-management programs or interventions on T2D among adult Asian Americans. Demographic characteristics are presented in Table 1. Majority of the studies (*n*=13, 72.2%) were conducted in or after 2013^15–17,19–21,23,25–27,29,31,32^ with no studies included before 2004. More than 66% are represented by East Asian Americans, namely more than one-third (*n*=8, 44.4%) on Chinese Americans^16,17,19,22,26,27,30,32^ followed by Korean Americans (*n*=4, 22.2%).^23–25,28^ Although not specifically focused on Chinese Americans, we categorized Le et al.'s^27^ study as Chinese American as the sample population was predominantly Chinese Americans. Only four subgroups from South and Southeast Asian Americans were represented: two studies (11.1%) were majority Filipino Americans,^15,29^ including Tomioka et al.'s study where the sample represented 92% Filipino Americans. Bangladeshi Americans^21^ and Hmong Americans^18^ were both represented in one study each. Two articles did not specify the Asian American subpopulation or nationalities.^20,31^

More than half of the studies took place in California or Hawaii (*n*=10, 55.6%), followed by Maryland or Washington, DC (*n*=5, 27.8%). Many of the studies that reported the gender of the participants (17 of the 18 citations) were conducted among female participants (>50% female; *n*=14, 82.4%), ranging from 41% to 100% female participants. Many of the participants were also older adults (mean=61.1 years old, standard deviation=5.3, range=53.4–73.0), and mostly had an education less than high school (*n*=11, 61.1%). For the 10 studies that reported participants' income level, majority earned less than $50,000 annually (*n*=8, 80%). Majority of the studies also had a concordance of the language spoken with one of the preferred languages of the subgroups (e.g., Chinese American study had Cantonese or Mandarin as a primary language).

#### Aims, framework, and research methods

##### Aims

The summary of the aims, framework, and design used in the 18 studies is in Table 2. More than half of the studies (*n*=10, 55.6%) tested the feasibility, efficacy, effectiveness, or acceptability of a self-management intervention. Of these 10 feasibility studies, four citations focused on Chinese Americans^19,22,30^ and Korean Americans^23,25,28^ while two were on Filipino Americans,^15,29^ and one citation on Bangladeshi Americans.^21^

**Table 2.** Aims, Publication Year, Theoretical/Conceptual Framework, and Research Design

Author	Asian American group representation	Aim	Framework	Design
Bender et al. (2017)	Filipino American	To evaluate the feasibility and efficacy of a culturally adapted weight loss lifestyle mobile health app.	Social Cognitive Theory and Transtheoretical Model for Health Behavior Change	RCT, Pilot
Chesla et al. (2014)	Chinese American	To examine differences between males and females in factors associated with diabetes management pre-and post-intervention.	Intersectionality	Single-cohort quasi-experimental (repeated measures) design
Chesla et al. (2013)	Chinese American	To develop culturally accessible diabetes for immigrant Chinese Americans using coping skills training to improve diabetes care processes and outcomes.	CBPR+Chinese Coping Skills Test Model	Single-cohort quasi-experimental with 4-month delayed treatment condition
Culhane-Pera et al. (2005)	Hmong American	To explore group visits' influence on diabetes management.	Chronic Care Model	Three groups quasi-experimental pre-post design
Ho et al. (2021)	Chinese American	To assess the acceptability, demand, and limited efficacy of an INC (a Chinese medicine and biomedicine based nutrition curriculum) for Chinese Americans with T2D.	Bowen's framework for testing feasibility	Convergent mixed-methods design
Ho et al. (2020)	Chinese American	To test the feasibility of INC intervention among Chinese Americans with T2D.	INC framework+culture-centered approach	Two-arm RCT
Inouye et al. (2015)	Asian American Pacific Islander	To investigate the effect of CBT intervention among Asian Americans with T2D.	Not mentioned	Double-blind RCT
Islam et al. (2013)	Bangladeshi American	To explore the feasibility and effect of a culturally and linguistically tailored CHW intervention among Bangladeshis with T2D in New York City.	CBPR	Single cohort quasi-experimental pre-post design
Ivey et al. (2012)	Chinese American	To assess the feasibility of implementing a Bodenheimer's teamlet model for diabetes care among Chinese American patients.	Bodenheimer's teamlet model	Two groups quasi-experimental pre-post design
Kim et al. (2016)	Korean American	To determine the effectiveness of a self-help intervention managed by nurses or CHWs among Korean Americans with T2D.	Self Help, PRECEDE-PROCEDE, RE-AIM	RCT
Kim et al. (2015)	Korean American	To compare the difference between nurses and CHW as counselors or case managers in diabetes outcomes among Korean Americans with T2D.	Self Help, PRECEDE-PROCEDE	RCT
Kim et al. (2009)	Korean American	To test the efficacy of a culturally tailored comprehensive diabetes management intervention for Korean Americans with T2D.	Not mentioned	RCT (pilot, delayed intervention)
Kwan et al. (2014)	Chinese American	To examine the link between social relationships and diabetes management support among Chinese Americans.	Contextual Adult Lifespan Theory for Adapting Psychotherapy	Single cohort quasi-experimental pre-post design
Le et al. (2013)	Chinese American (majority—77%)	To examine the effectiveness of a culturally-tailored diabetes pilot clinic for Asian Americans in reaching the glycemic target.	Not mentioned	Observational—retrospective study
Song et al. (2010)	Korean American	To describe the process of translating evidence-based dietary guidelines into a tailored nutrition education program for Korean Americans with T2D.	CBPR, American Diabetes Education, and Korean Diabetes guidelines	RCT (delayed intervention)
Tomioka et al. (2014)	Filipino American (majority—92%)	To test the 6-month impact of Stanford's DSMP adapted for AAPI on behavioral and clinical indicators.	Not mentioned	Single cohort pre-post quasi-experimental design
Wang and Chan (2005)	Chinese American	To determine the feasibility and acceptability of a tailored culturally appropriate T2D management program among Chinese Americans and the preliminary outcomes of the intervention.	Empowerment model	Single cohort pre-post quasi-experimental design
Yomogida et al. (2015)	Asian Americans	To examine the effects of a cognitive-behavioral intervention on diet and exercise among AAPI with T2D.	Not mentioned	Doubled blind, two-arm RCT

AAPI, Asian Americans and Pacific Islanders; CBPR, Community-Based Participatory Research; CBT, cognitive behavioral therapy; CHW, community health worker; DSMP, Diabetes Self-Management Program; INC, integrative nutritional counseling; PRECEDE, Predisposing, Reinforcing, and Enabling Constructs in Educational Diagnosis and Evaluation; PROCEDE, Policy, Regulatory, and Organizational Constructs in Educational and Environmental Development; RCT, randomized controlled trial; RE-AIM, reach, effectiveness, adoption, implementation, maintenance; T2D, type 2 diabetes.

Three studies^21,23,25^ (16.7%) examined the impact of community health workers (CHWs) on diabetes management. Another three studies^18,22,26^ explored the impact of group, social network, or team model on individuals' diabetes self-care activities or management. Two different studies explored group variances in participants and interventionists; one was on gender differences of participants associated with diabetes management^17^ while another was on the differences between nurses and CHWs as interventionists in diabetes outcomes.^25^ Three studies explored the feasibility of a nutrition-related^19,28,32^ or weight-loss intervention^15^ on diabetes outcomes, while another three studies examined the effects of cognitive or behavioral interventions.^16,20,31^

##### Framework

Thirteen (72.8%) of the 18 citations included a conceptual model or framework to ground their study. Three studies used Community-Based Participatory Research (CBPR)^16,21,28^; two used the self-help and PRECEDE-PROCEDE (Predisposing, Reinforcing, and Enabling Constructs in Educational Diagnosis and Evaluation - Policy, Regulatory, and Organizational Constructs in Educational and Environmental Development).^23,25^ Other models used included the Chronic Care Model, Empowerment Model, and Contextual Adult Lifespan Theory of Adapting Psychotherapy.

##### Design

Overall, eight studies (44.4%) used randomized controlled trials as their research method,^15,19,20,23–25,28,31^ while another eight were quasi-experimental/pre–post design.^16–18,21,22,26,29^ One study (5.6%) used an observational retrospective study^27^ and one used a convergent mixed-methods study design.^32^

#### Intervention typology

##### Intervention types, delivery modalities, and interventionists

Table 3 displays the intervention typology of the citations, including the interventionists, delivery modalities, and description of the control arm (if applicable).

**Table 3.** Intervention Typology: Type of Intervention, Interventionists, Delivery Modality, and Control Arm Intervention

Author	Asian American group representation	Type of intervention	Interventionists	Intervention delivery	Control arm intervention (if applicable)
Bender et al. (2017)	Filipino Americans	Weight loss intervention, lifestyle education, and coaching	Trained research staff	Technology-based	Active Waitlist Control Group (received intervention in Phase 2)
Chesla et al. (2014)	Chinese Americans	DSME in Cantonese	Trained Interventionist	In-person	N/A
Chesla et al. (2013)	Chinese Americans	Cognitive Behavioral Treatment	Workgroup members who were bilingual (Cantonese and English) social workers, health educators, counselors with past training in behavioral methods, and extensive experience in the care of Chinese immigrants	In-person	
Culhane-Pera et al. (2005)	Hmong Americans	Culturally-familiar Group visit	group discussions were led by Hmong health care professionals in Hmong: clinic family doctor, Hmong diabetes nurse educator, Hmong social worker, Hmong exercise specialist	In-person	Usual diabetes care and refusers
Ho et al. (2021)	Chinese Americans	Integrative Nutrition Counseling+Biomedical nutrition concepts	Internal medicine physicians, licensed acupuncturists/medical anthropologist, registered dietitian, health communication scholar, linguist	In-person	N/A
Ho et al. (2020)	Chinese Americans	DSME+Integrative Nutrition	RN diabetes educator, acupuncturist, biomedical providers, Chinese medicine providers	In-person	Usual DSME
Inouye et al. (2015)	Asian Americans	CBT	Research assistants trained in CBT	In-person	DES
Islam et al. (2013)	Bangladeshi Americans	Self-help intervention (adapted from existing curricula materials validated in minority communities)	Two trained bilingual Bangladeshi CHWs (one male, one female)	In-person	N/A
Ivey et al. (2012)	Chinese Americans	Ethnic and language-concordant teamlet model by using health coaches tailored for Chinese patients	MD visit, registered dietitian, health coaches	Blended (in-person and telephone-based)	Usual care
Kim et al. (2016)	Korean Americans	Self-help intervention	CHWs and RNs	Blended	Usual care
Kim et al. (2015)	Korean Americans	Community-based multi-modal behavioral SHIP-DM	CHWs and registered nurses	In-person	Delayed intervention after month 12
Kim et al. (2009)	Korean Americans	Community-based, culturally tailored behavioral intervention	Self, nurse, nutritionist	Blended approach	Delayed intervention
Kwan et al. (2014)	Chinese Americans	Culturally-adapted diabetes intervention	Not reported	In-person	N/A
Le et al. (2013)	Chinse Americans	Linguistic and cultural knowledge of staff with culturally appropriate diabetes management program (clinic)	Asian Clinic team at Joslin: endocrinologists, dietitian and diabetes educator, care coordinator, exercise physiologist, medical assistants, nurse practitioners	Blended (in-person+the use of the interactive website)	Adult Diabetes Clinic (Usual Care)
Song et al. (2010)	Korean Americans	Culturally-tailored Nutritional education program (self-help intervention)	Bilingual dietician, study participants, their family members, community health workers, and diabetes educators	In-person	Delayed intervention
Tomioka et al. (2014)	Filipino Americans	DSMP	Physician, DSMP facilitators	In-person	N/A
Wang and Chan (2005)	Chinese Americans	Culturally-tailored Diabetes Management Intervention program (dietary education, exercise, self-care, medication)	Investigator and registered nurse	In-person	N/A
Yomogida et al. (2015)	Asian Americans	Cognitive Behavioral intervention	Program coordinator or research assistants led the sessions and were supervised by a highly experienced researcher of diabetes-related clinical trials	In-person	Usual care

DES, Diabetes Education and Support; DSME, Diabetes Self-Management Education; N/A, not applicable; RN, registered nurse; SHIP-DM, self-help intervention program for diabetes management.

The interventions were grouped into five different types: diabetes self-management education (*n*=7),^17,19,26,27,29,30,32^ cognitive behavioral therapy (CBT; *n*=4),^16,24,31^ self-help (*n*=4),^21,23,25,28^ group-type intervention (*n*=2),^18,22^ and weight-specific intervention (*n*=1).^15^

For Chinese Americans, most of the interventions were related to self-management education (*n*=6), followed by one CBT and group-type intervention. Korean American studies were mostly about self-help interventions (*n*=3) with one related to CBT. Filipino-Americans had one intervention on self-management education and another on weight-based intervention.

Overall, most of the interventions were delivered in-person (*n*=13, 72.2%),^16–21,25,26,28–32^ four studies (22.2%) with a blended approach (combination of in-person and technology),^22–24,27^ and one study (5.6%) with a technology-only intervention.^15^ Many of the interventionists were licensed health care professionals (*n*=12, 66.7%), including dietitians/nutritionists (*n*=5, 27.8%), nurses (*n*=8, 44.4%), physicians (*n*=7, 38.9%), diabetes educators (*n*=6, 33.3%), exercise specialists (*n*=2, 11.1%), acupuncturists (*n*=2, 11.1%), health coaches (*n*=1, 5.6%), and medical assistants (*n*=1, 5.6%). Researchers or trained interventionists were also commonly used (*n*=8, 44.4%), followed by community health workers (*n*=4, 22.2%) and social workers (*n*=1, 5.6%). One study^26^ did not report the interventionists involved in their study.

Supplementary Appendix SA2 has more comprehensive information showing the (1) description of the intervention and control arms (if applicable), (2) primary, secondary, or other outcomes, and (3) outcome values for both intervention and control arms. Supplementary Appendix SA3 displays the different components available to abstract and their corresponding codes derived from the DSME/S Joint Position Statement and ADA DSEM/S Standards. All the studies that reported the individual components of their interventions (17 of the 18 citations) included Assessment and education (e.g., some form of assessment of cultural beliefs, T2D management basics, attitudes, knowledge assessment, etc.).

Other major components that were included in these interventions or programs included developing some personal strategies to combat psychosocial issues (*n*=13, 72.2%),^15–18,20–25,28–31^ strategies to promote health and behavior changes (*n*=12, 66.7%),^15,17,19–25,27,30,31^ and nutrition (*n*=11, 61.1%).^15,19,21–25,28,30–32^ Explicit risk reduction component of a program or intervention was seen in only two studies (11.1%).^25,30^

##### Cultural adaptation strategies

Table 4 displays the intervention types and the summary of how each study tailored its intervention strategies. We based the groupings on the cultural adaptation guidelines recommended by Kreuter et al. with the following dimensions: (1) linguistics, (2) peripheral, (3) evidential, (4) constituent-involving, and (5) sociocultural.^33^ Linguistic techniques aim to make materials or programs more accessible to the target community by using the participants' preferred language. Peripheral strategies try to appeal to the participants by using specific colors, graphics, fonts, declarative titles, or portraits of group members. Evidential strategies use evidence to influence the group. Constituent-involving techniques are approaches that depend primarily on the expertise or experience of the target group. Sociocultural strategies apply a group's cultural values, beliefs, and behaviors to provide context and meaning to information and messages.

**Table 4.** Interventions: Cultural Adaptation Strategies Using Kreuter et al.'s Categories

Author	Type of intervention	Linguistics	Peripheral	Evidential	Constituent-involving	Sociocultural
Bender et al. (2017)	PilAm Go4Health	Lifestyle education pamphlets translated in Tagalog.	Photos of common Filipino foods were used in pamphlets.	Sessions contained information on prevalence and factors associated with T2D among Filipino Americans.	Community stakeholders (e.g., leaders, members, health providers) helped with study design.	Family members attended scheduled office-visits.
Chesla et al. (2014)	DSME in Cantonese	Cultural communication strategies (e.g., skilled indirect communication; resolving conflict in Chinese or American cultural contexts). Cultural idioms and stories. Handouts translated into Cantonese with community expressions and norms.	Not reported.	Collective rather than individual problem-solving strategies. Diabetes self-management review presented by a skilled, Cantonese-speaking certified Diabetes Educator.	Qualitative study to assess unique needs, community partnership to adapt the treatment, pilot testing with strong feedback.	Interventionists were work group members and bilingual with extensive experience in care of Chinese immigrants.
Chesla et al. (2013)	Cognitive Behavioral Treatment	Cultural communication strategies (e.g., skilled indirect communication; resolving conflict in Chinese or American cultural contexts). Cultural idioms and stories. Handouts translated into Cantonese with community expressions and norms.	Not reported.	Collective rather than individual problem-solving strategies. Diabetes self-management review presented by a skilled, Cantonese-speaking certified Diabetes Educator.	Qualitative study to assess unique needs, community partnership to adapt the treatment, pilot testing with strong feedback.	Interventionists were work group members and bilingual with extensive experience in care of Chinese immigrants. Family communication strategies and conflict resolution.
Culhane-Pera et al. (2005)	Culturally familiar Group visit	Group discussions led by Hmong health care professional in Hmong focused on a topic of diabetes management.	Exploration of participants' beliefs, experiences and attitudes about healthy behaviors (specifically diet, group exercise, and medications).	Group discussions' format was informal and interactive (nonauthoritative), encouraging adoption of diabetes management strategies, problem solving, goal setting and sharing experiences.	Not reported.	Personnel included a Hmong DM nurse educator, Hmong research assistant, Hmong medical assistant, Hmong social workers, and Hmong exercise specialist. Family encouraged to participate.
Ho et al. (2021)	Integrative nutrition counseling (INC) + biomedical nutrition concepts	Booklet printed in Chinese traditional characters and English.	INC uses Chinese and English wording (e.g., Chinese foods as examples and in pictures). Culturally appropriate guidelines including practical tips and diet recommendations using Chinese Medicine principles.	INC guideline.	INC developed with input from biomedical providers, Chinese medicine practitioners, and Chinese Americans with T2D.	Team members with expertise working in Chinese and Chinese American communities. Diagnostic interview with acupuncturist.
Ho et al. (2020)	DSME+Integrative Nutrition	INC guidebook in English/Chinese.	Chinese medicine diagnosis and learned about nutrition (integration of Chinese medical food principles). INC guidebook contained food list and pictures.	Followed INC guideline.	INC developed with input from biomedical providers, Chinese medicine practitioners, and Chinese Americans with T2D.	Met with acupuncturist who used diagnostic techniques typical of traditional Chinese medicine.
Inouye et al. (2015)	CBT	Not reported.	Not reported.	Not reported.	Not reported.	Family members or supportive friend were encouraged to attend and participate.
Islam et al. (2013)	Self-help intervention (adapted from existing curricula materials validated in minority communities)	Group session visits were conducted in Bengali held in clinical and community settings. All materials were developed in English translated in Bengali by certified translator and reviewed by CHWs for accuracy.	Culturally tailored group activities, physical exercise, diet and foods, and gender-specific exercises (e.g., healthy options for ghee, chai tea; Bengali alternatives to high fat desserts; review of yoga and tai-chi exercises).	Adapted the DREAM curricula.	Community partners and CHWs involved in the development of the intervention (from formulation of research question, data collection, retention).	Intervention delivered by two trained, bilingual Bangladeshi CHWs (community leaders) in locations convenient to participants (e.g., home, community locations, restaurants, clinics). Family members encouraged to participate.
Ivey et al. (2012)	Teamlet model	Language appropriate diabetes education materials.	Not reported.	Physician and health coach visit, teamlet huddle, follow-up visit.	Community-academic partnership. An advisory committee (composed of patients, family members, professionals from community with diabetes expertise or work within the community) informed the design, implementation, interpretation and dissemination of results.	Health coaches, dietitian, and most physicians were ethnically and linguistically matched to Chinese participants. They used clinic interpreter for physicians who did not speak patient's language. Follow-up with patient and/or family member for support.
Kim et al. (2016)	Self-help intervention	Not reported.	Not reported.	Not reported.	Korean American community-academic partnership.	CHWs reside in Korean American community.
Kim et al. (2015)	Community-based multi-modal behavioral SHIP-DM	Not explicitly reported but expected that it was tailored to Korean Americans.	Not reported.	Brochure contained critical self-management principles of SHIP-DM, available care and educational resources in the community.	Not reported.	Motivational interviewing by a team of bilingual nurses/CHWs with extensive training in diabetes management.
Kim et al. (2009)	Community-based culturally tailored behavioral intervention (SHIP-DM)	Not reported.	Not reported.	Not reported.		Structured education program delivered at a community site by trained bilingual nurses and nutritionist.
Kwan et al. (2014)	Culturally adapted diabetes intervention	Not reported.	Not reported.	Not reported.	Not reported.	Not reported.
Le et al. (2013)	Linguistic and cultural knowledge of staff with culturally appropriate diabetes management program (clinic)	Linguistic and cultural knowledge of the staff along with culturally appropriate educational materials (e.g., most educational materials are available in the patients' primary languages).	Dietitian familiar with many Asian cuisines and offered culturally tailored self-care education and training.	Includes endocrinologists, a dietitian who is also a certified diabetes educator, and a care coordinator, all of whom are skilled in integrating the unique physiology, cultural beliefs, and explanatory models of predominant Asian cultures into the care they provide. All educational materials follow the diabetes center's guidelines.	Not reported.	Cultural knowledge of the staff along with culturally appropriate educational materials. May include family members.
Song et al. (2010)	Culturally tailored Nutritional education program (self-help intervention)	Education classes offered in preferred language (Korean or English). Educational content translated from existing dietary guidelines.	Nutrition lectures addressed variety of nutrition topics using examples of traditional Korean foods; problem solving discussed Korean specific food preparation methods.	Guided by ADA's dietary guidelines.	Not reported.	Education classes delivered in a culturally relevant context to participants and family members.
Tomioka et al, (2014)	DSMP (Community clinic-based program)	Key messages in participants' native languages.	Not reported.	Not reported.	Partnership with community (Elder Care Center), Health Department, and Academic institution.	Pre-workshop orientation by program leaders and Elder Care Center physician; graduation ceremony which family members were invited.
Wang and Chan (2005)	Culturally tailored Diabetes Management Intervention program	Certified diabetes educator taught group sessions in Mandarin, Cantonese or Taiwanese. Handouts of lecture notes in Chinese.	Cultural values in dietary practice (e.g., explain Chinese diet patterns), exercise (e.g., pros and cons of common Chinese exercises), medication (e.g., pros and cons of Chinese and Western medicine) integrated.	Cultural values in diabetes self-related self-care behaviors were integrated.	Not reported.	Sessions taught in Mandarin, Cantonese or Taiwanese. Family members were encouraged to be involved in the learning process.
Yomogida et al. (2015)	Cognitive behavioral intervention	Not reported.	Use of Nutritionist Pro (that consists of over 35,000 different foods including foods unique to Hawai'i).	Not reported.	Not reported.	Cultural influences on behaviors. Spouse/partner was encouraged to participate in the sessions.

ADA, American Diabetes Association; DM, diabetes mellitus; DREAM, Diabetes Research, Education, and Action for Minorities; INC, integrative nutritional counseling.

*Source:* Kreuter et al.^33^

Majority of the studies used linguistic and sociocultural strategies such as using language-appropriate materials (e.g., translating diabetes materials into the participants' preferred languages), conducting sessions in participants' language, culturally tailoring exercises, involving community stakeholders, and utilizing community group members as interventionists. Although many of the studies encouraged family members to participate,^18,20,21,27,29–31^ only a limited number of studies capitalized family or family members as a central part of the intervention.^15,16,22,28^

#### Outcomes

##### Categorized outcome measures

**FIG. 2.**
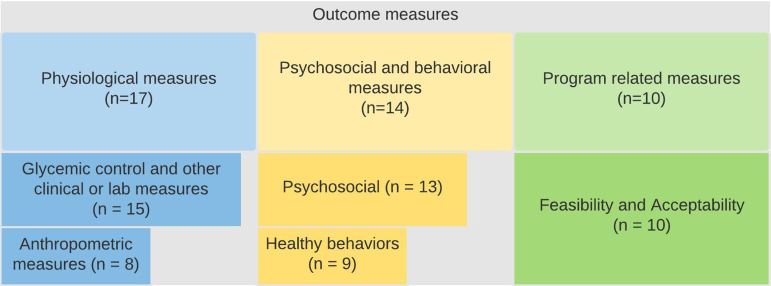
Categorized outcome measures.

We organized the outcome measures (Fig. 2) into the following three major themes: (1) physiological, (2) psychosocial and behavioral, and (3) program-related outcomes. Physiological outcomes were further divided into two subcategories: (1a) glycemic control and other clinical or laboratory measures, and (2a) anthropometric measures. Psychosocial and behavioral outcomes were also divided into two subcategories: (2a) healthy behaviors and (2b) psychosocial outcomes.

Except for three studies,^17,31,32^ all citations measured some form of a physiological outcome. Majority of the studies (*n*=14, 77.8%) measured glycemic control or clinical/laboratory measurements^15,16,18–27,29,30^ or anthropometric measurements (*n*=8, 44.4%).^15,18,20,21,24,29,30,32^ Psychosocial and behavioral outcomes included healthy behaviors (*n*=8, 44.4%)^15,18,19,21,24,29,31,32^ or psychosocial outcomes (*n*=12, 66.7%).^16–21,23–25,28,29,32^ More than half of the studies (*n*=11, 55.6%) also included program-related outcomes, measuring the feasibility or acceptability of the participants' interventions or fidelity and satisfaction.^15,16,19,21–23,25,28–30,32^

##### Differences in outcome measures

Table 5 presents the studies' outcome measures categorized by specific measures, authors, and subpopulations. We used three-shaped icons to indicate either an improvement, no change, or decline of an outcome from baseline to the end of the measurement. That is, a green circle indicates an improvement of an outcome, an amber triangle indicates no change, and a red diamond indicates a decline in the outcome measure from baseline to final measurement. For studies that had a control arm, Table 5 only represents a comparison of the intervention arm; therefore, dashboard displays a change from the baseline measure to the final measure of the intervention group. Supplementary Appendix SA2 has information of the control arm measurements.

**Table:** 

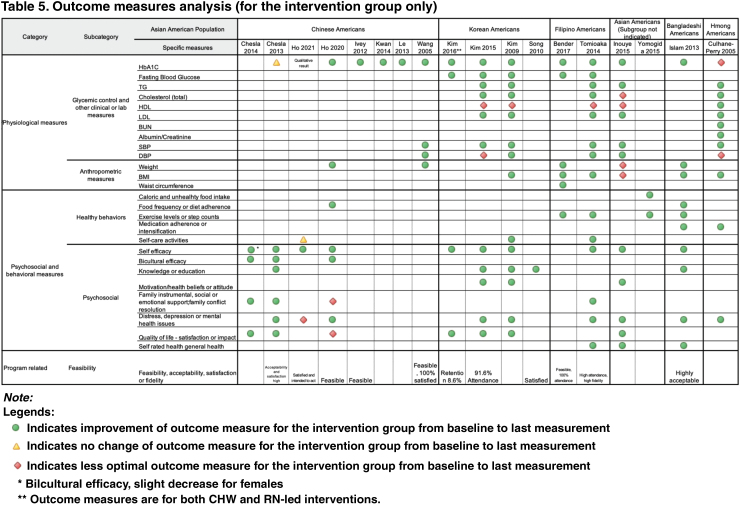

Overall, we saw improvements in physiological measures in most of the studies. Of the 15 studies, 12 or 80.0% showed an improvement in the participants' HbA1C measure. Two studies (on Chinese- and Hmong Americans)^16,18^ showed no improvement or an increase in HbA1C. For fasting blood glucose, 100% of all five studies showed a decrease at the end of the measurement period. Three of the studies were on Korean Americans, and two were on Filipino Americans. Similarly, most anthropometric measurements such as weight and body mass index (BMI) showed a reduction in their respective end measurements.

For healthy behaviors such as exercise intensification and self-care activities, most studies showed an improvement in these behaviors. Likewise, studies that measured efficacy, knowledge, attitude, motivation, quality of life, or general health showed improvement from baseline. Similarly, most of the studies that measured distress or depression showed reduced symptoms, suggesting improvement after participating in a self-management intervention or program.

## Summary of Evidence

This scoping review identified 18 primary studies addressing T2D self-management interventions among Asian Americans in the United States published between 2005 and 2021. Most of these studies included a framework to guide their work. Many of the studies' intervention components had the elements recommended by the ADA, from current knowledge assessment to strategies to promote behavior change. The outcomes included physiological and psychosocial behaviors. Many of the interventions that assessed program-related outcomes were considered feasible and acceptable.

### Gaps in current literature

In this scoping review, we found several gaps in the current literature of T2D self-management interventions among Asian Americans. We focused our discussion on three areas: underrepresented Asian Americans, gender and geographic disparities, and the lack of technology-infused interventions.

Despite the growing population of Asian Americans in the United States^34^ and the increasing prevalence of diabetes among this group,^1^ the relatively small number of research studies on this issue suggest a lack of targeted T2D self-management interventions across various subpopulations of Asian Americans. Specifically, we found a paucity of studies on South Asian Americans and Southeast Asian Americans. Most of the studies included East Asian Americans (i.e., Chinese Americans and Korean Americans). This is particularly notable as the age- and sex-adjusted prevalence of diabetes among South Asians and Southeast Asians are 23.3% (95% confidence interval [CI] 15.6–30.9) and 22.4% (95% CI 15.9–28.9%), respectively.^6^ Considering focused interventions in understudied Asian American subpopulations may be warranted.

While none of the studies focused exclusively on a specific gender, most studies had majority of female study samples. This is similar to the gender patterns of participants in other studies on chronic disease self-management education programs. For instance, Smith et al. found that males have low participation in self-management programs compared to females.^35^ Some potential challenges include scheduling issues, job and family responsibilities, personal relevance, in-person versus online availability.^35^ In addition, gender is a spectrum—and it is not only male or female. Some individuals may identify as nonbinary, gender diverse, or gender nonconforming. As previous studies have reported gender differences in self-management needs,^36,37^ collecting gender data that are not dichotomous and instead reflect fluidity and targeted gender recruitment may be warranted among Asian Americans.

We found evidence of geographic disparities in the studies included in our review. Most of the studies were conducted in California and Hawaii, an expected pattern as these two states have one of the largest Asian American populations.^34^ However, other states such as New York, Texas, New Jersey, Illinois, Florida, Virginia, and Massachusetts have a large or growing population of Asian Americans and were under or not represented in the studies. Expanding recruitment to include Asian Americans in these states may be needed to improve generalizability and increase diversity and representation.

The bulk of the interventions were implemented in person. Only five studies included some form of technology-enabled intervention, including a blended approach of in-person and technology. As self-management continues to evolve to have new and emerging delivery modalities such as telehealth, this presents an opportunity for researchers and community organizers to introduce technology in self-management among Asian Americans by exploring the feasibility of technology in specific Asian American subgroups. In two different literature reviews, authors found that technology-enabled programs positively impacted individuals' self-management and self-care behaviors^38,39^ and that technology-based diabetes studies in minority populations are highly needed.^38^ Innovative use of technology such as digital phenotyping and data visualization is necessary to help enhance and sustain technology-enabled self-management strategies.^40,41^

### Improvement in health outcomes

Across the different subgroups of Asian Americans represented in this study, we saw an overall improvement in many physiological measures, particularly decreased HbA1C levels, cholesterol, weight, and BMI. We also saw improvement in healthy behaviors, such as an increase in exercise levels and self-care activities. Similarly, some psychosocial measures such as self-efficacy, diabetes knowledge, and quality of life improved after self-management interventions. These outcomes are consistent with the benefits outlined by the American Association of Diabetes Educators and the Academy of Nutrition and Dietetics on self-management programs.^8^

Although many of the studies included in this review improved self-efficacy, we highlight bicultural efficacy as an essential outcome measure among Asian Americans. Bicultural efficacy is the belief, confidence, ability, or perceived expectations to handle challenges living in two cultures without compromising culture or self-identity.^42^ This is critical, as studies have shown a positive association between bicultural efficacy with Asian Americans' health, as demonstrated in a meta-analysis of 141 studies.^43^ With the cultural and health care challenges faced by Asian Americans, examining how to maintain family and social relationships, utilizing the health care system in the United States, and dealing with language and different lifestyles needs to be incorporated more in future programs and interventions.

Although nutrition was a major component in many self-management interventions, only three studies explored nutrition, food intake, or diet adherence among Asian Americans to diabetes outcomes. Nutrition is critical as Asian Americans are prone to developing T2D even at lower body weights.^44^ As a healthy diet is essential in the management of diabetes, and food plays an integral part in the culture of Asian Americans, future studies examining how food or food intake by race or ethnicity could inform the development of culturally appropriate interventions or programs.

#### Culturally adapted interventions

For interventions that have shown improvement in physiological, psychological, and behavioral outcomes, we found common themes implemented in these interventions. Many translated educational materials to participants' preferred language, culturally appropriate materials, and had staff's linguistic and cultural concordance. Some interventions also used community stakeholders to design the interventions, and interventionists were part of the community.

### Limitations

This review has several limitations. This study excluded other types of diabetes, and we acknowledge that many interventions or programs are not exclusively on T2D; therefore, some studies that implemented interventions of T2D with a combination of other types of diabetes were not included. Although not intentional, many qualitative studies were excluded in this study that may have provided a richer and more diverse understanding of the individuals' experience with T2D self-management. Although our search strategy was sensitive, we excluded literature outside of English.

## Conclusions

Although there was heterogeneity in the different aims, framework, components, and outcomes in examining various self-management interventions or programs on Asian Americans, there were also some similarities. Most of these studies included a framework to guide their work, and many of the interventions were culturally tailored. Many of the components of the interventions had the elements recommended by the ADA, such as assessment of current knowledge, medication, personal strategies to promote health and behavior change, and others. The majority of the studies measured outcomes such as HbA1C and self-efficacy. Most of the interventions were considered feasible, acceptable, showed high attendance, and satisfied individuals with the programs. Educational materials and culturally appropriate sessions, availability in participants' primary language, familiarity with the culture, and culturally tailored self-care and education were factors in observing changes in outcomes.

As we identified several gaps in the literature, we recommend future research considerations that include a health equity framework in grounding intervention studies, the diversification of interventionists, targeted recruitment of men, and engaging the community and other Asian American stakeholders in developing T2D self-management interventions.

## Supplementary Material

Supplemental data

Supplemental data

Supplemental data

## Supplementary Material

Supplementary Appendix SA1

Supplementary Appendix SA2

Supplementary Appendix SA3

## Abbreviations Used

AAPI: Asian Americans and Pacific Islanders
ADA: American Diabetes Association
BMI: body mass index
CBPR: Community-Based Participatory Research
CBT: cognitive behavioral therapy
CHW: community health worker
CI: confidence interval
DES: Diabetes Education and Support
DREAM: Diabetes Research, Education, and Action for Minorities
DSME: Diabetes Self-Management Education
DSME/S: Diabetes Self-Management Education and Support
DSMP: Diabetes Self-Management Program
INC: integrative nutritional counseling
PRECEDE: Predisposing, Reinforcing, and Enabling Constructs in Educational Diagnosis and Evaluation
PRISMA: Preferred Reporting Items for Systematic reviews and Meta-Analyses
PROCEDE: Policy, Regulatory, and Organizational Constructs in Educational and Environmental Development
RCT: randomized controlled trial
RE-AIM: reach, effectiveness, adoption, implementation, maintenance
SHIP-DM: self-help intervention program for diabetes management
T2D: type 2 diabetes
