# Supplementary material for: Type 2 Diabetes Self-Management Interventions Among Asian Americans in the United States: A Scoping Review
Source: Health Equity. 2022 Sep 23;6(1):750–66. doi: 10.1089/heq.2021.0083 (PMC9536350; doi:10.1089/heq.2021.0083)
Supplement: Supplemental data [file Suppl_AppSA1.docx]

**Supplementary Appendix 1: Search Strategies**

**Medline (OVID)**

**Date searched:**  December 7, 2020
**Number of Results:** 996

**Date searched:** June 17, 2021
**Number of results:** 36
**Limited to:** dt.=20200707-20210617

1. Asian Americans/

2. ((asia* or korea* or chinese or china or vietnam* or hmong or filipino* or indian* or japan* or burm* or nepal* or bangladesh* or indonesia* or pakistan* or malaysia* or myanmar or cambod* or singapor*) adj3 american*).ti,ab,kw.

3. 1 or 2

4. exp Diabetes Mellitus, Type 2/ or insulin resistance/

5. "diabet*".ti,ab,kw.

6. (type2 or T2D or T2DM or "type 2" or "type-2" or "type II" or "type-II" or TIID or NIDDM or "NonInsulin Dependent" or "Non-Insulin Dependent" or "Adult-Onset" or "insulin resistance").ti,ab,kw.

7. 4 or 5 or 6

8. Self-Management/

9. exp Self Care/

10. exp Health Behavior/

11. exp Self-Help Devices/

12. exp disease management/

13. exp Exercise/

14. exp Technology/

15. exp Life Style/

16. exp Health Education/

17. exp Behavior Therapy/

18. exp Telemedicine/

19. exp Counseling/

20. exp Self-Control/

21. self efficacy/

22. problem solving/

23. exp decision making/

24. Health Knowledge, Attitudes, Practice/

25. ((problem adj3 solving) or (decision adj3 making)).ti,ab,kw.

26. ((self or symptom or medical or disease or emotional) adj3 (care or control or efficacy or administ* or monitor* or manage* or medicat* or help or regulat*)).ti,ab,kw.

27. ((obes* or family or pain or weight or individual) adj3 (manage* or program* or reduc*)).ti,ab,kw.

28. (cultur* adj3 (appropriate or tailor* or relevan* or adapt*)).ti,ab,kw.

29. (exercis* or "physical activit*" or "life style" or lifestyle or "Diabetes Prevention Program" or DSMP or DPP or DSME* or DSMS or technol* or educat* or counsel* or coach* or "health behavior*" or therap* or technolog* or telemedicine or telehealth).ti,ab,kw.

30. 8 or 9 or 10 or 11 or 12 or 13 or 14 or 15 or 16 or 17 or 18 or 19 or 20 or 21 or 22 or 23 or 24 or 25 or 26 or 27 or 28 or 29

31. 3 and 7 and 30

**Scopus**

**Date searched:**  December 7, 2020
**Number of Results:** 1748

**Date searched:** June 17, 2021
**Number of results:** 32
**Date filter:** 2021

**#1:**

TITLE-ABS-KEY( ( asia*  OR  korea*  OR  chinese  OR  china  OR  vietnam*  OR  hmong  OR  filipino*  OR  indian*  OR  japan*  OR  burm*  OR  nepal*  OR  bangladesh*  OR  indonesia*  OR  pakistan*  OR  malaysia*  OR  myanmar  OR  cambod*  OR  singapor* )  W/3  american* )

**#2**

TITLE-ABS(diabet* OR type2 OR T2D OR T2DM OR "type 2" OR "type-2" OR "type II" OR "type-II" OR TIID OR NIDDM OR "NonInsulin Dependent" OR "Non-Insulin Dependent" OR "Adult-Onset" OR "insulin resistance" )

**#3**

TITLE-ABS-KEY ( ( ( problem  W/3  solving )  OR  ( decision  W/3  making ) )  OR  ( ( self  OR  symptom  OR  medical  OR  disease  OR  emotional )  W/3  ( care  OR  control  OR  efficacy  OR  administ*  OR  monitor*  OR  manage*  OR  medicat*  OR  help  OR  regulat* ) )  OR  ( ( obes*  OR  family  OR  pain  OR  weight  OR  individual )  W/3  ( manage*  OR  program*  OR  reduc* ) )  OR  ( cultur*  W/3  ( appropriate  OR  tailor*  OR  relevan*  OR  adapt* ) )  OR  exercis*  OR  "physical activit*"  OR  "life style"  OR  lifestyle  OR  "Diabetes Prevention Program*"  OR  dsmp  OR  dpp  OR  dsme*  OR  dsms  OR  technol*  OR  educat*  OR  counsel*  OR  coach*  OR  "health behavior*"  OR  therap*  OR  technolog*  OR  telemedicine  OR  telehealth )

**#4:**

(#1 AND #2 AND #3)

**Cochrane**

**Date searched:**  December 7,2020
**Number of Results:** 208

**Date searched:**  June 18,2021
**Number of Results:** 14

**Date added to Cochrane trials:** 07/12/2020-18/06/2021

**#1:**

[mh "Asian Americans"] OR ((asia* OR korea* OR chinese OR china OR vietnam* OR hmong OR filipino* OR indian* OR japan* OR burm* OR nepal* OR bangladesh* OR indonesia* OR pakistan* OR malaysia* OR myanmar OR cambod* OR singapor*) NEAR/3 american*):ti,ab,kw

**#2:**

[mh "Diabetes Mellitus, Type 2"] OR [mh "insulin resistance"] OR diabet*:ti,ab OR (type2 OR T2D OR T2DM OR "type 2" OR type-2 OR "type II" OR type-II OR TIID OR NIDDM OR "NonInsulin Dependent" OR "Non-Insulin Dependent" OR Adult-Onset OR "insulin resistance"):ti,ab,kw

**#3**:

[mh “Self-Management”] OR [mh "Self Care"] OR [mh "Health Behavior"] OR [mh "Self-Help Devices"] OR [mh "disease management"] OR [mh “Exercise”] OR [mh Technology] OR [mh "Life Style"] OR [mh "Health Education"] OR [mh "Behavior Therapy"] OR [mh Telemedicine] OR [mh Counseling] OR [mh "Self-Control"] OR [mh "self efficacy"] OR [mh "problem solving"] OR [mh "decision making"] OR [mh "Health Knowledge, Attitudes, Practice"] OR  ((problem NEAR/3 solving) OR (decision NEAR/3 making)):ti,ab,kw OR ((self OR symptom OR medical OR disease OR emotional) NEAR/3 (care OR control OR efficacy OR administ* OR monitor* OR manage* OR medicat* OR help OR regulat*)):ti,ab,kw OR ((obes* OR family OR pain OR weight OR individual) NEAR/3 (manage* OR program* OR reduc*)):ti,ab,kw OR (cultur* NEAR/3 (appropriate OR tailor* OR relevan* OR adapt*)):ti,ab,kw OR (exercis* OR "physical activit*" OR "life style" OR lifestyle OR "Diabetes Prevention Program" OR DSMP OR DPP OR DSME* OR DSMS OR technol* OR educat* OR counsel* OR coach* OR "health behavior*" OR therap* OR technolog* OR telemedicine OR telehealth):ti,ab,kw

**#4:**

#1 AND #2 AND #3

**CINAHL**

**Date searched:**  December 7, 2020
**Number of Results:** 630

**Date searched:** June 17, 2021
**Number of results:** 12
**Date filter:** 2021

**#1:**

(MH "Asians") OR (((TI asia* OR AB asia*) OR (TI korea* OR AB korea*) OR (TI chinese OR AB chinese) OR (TI china OR AB china) OR (TI vietnam* OR AB vietnam*) OR (TI hmong OR AB hmong) OR (TI filipino* OR AB filipino*) OR (TI indian* OR AB indian*) OR (TI japan* OR AB japan*) OR (TI burm* OR AB burm*) OR (TI nepal* OR AB nepal*) OR (TI bangladesh* OR AB bangladesh*) OR (TI indonesia* OR AB indonesia*) OR (TI pakistan* OR AB pakistan*) OR (TI malaysia* OR AB malaysia*) OR (TI myanmar OR AB myanmar) OR (TI cambod* OR AB cambod*) OR (TI singapor* OR AB singapor*)) N3 (TI american* OR AB american*))

**#2:**

(MH "Diabetes Mellitus, Type 2") OR (MH "insulin resistance") OR (TI diabet*,kw. OR AB diabet*,kw.) OR ((TI type2 OR AB type2) OR (TI T2D OR AB T2D) OR (TI T2DM OR AB T2DM) OR (TI "type 2" OR AB "type 2") OR (TI type-2 OR AB type-2) OR (TI "type II" OR AB "type II") OR (TI type-II OR AB type-II) OR (TI TIID OR AB TIID) OR (TI NIDDM OR AB NIDDM) OR (TI "NonInsulin Dependent" OR AB "NonInsulin Dependent") OR (TI "Non-Insulin Dependent" OR AB "Non-Insulin Dependent") OR (TI Adult-Onset OR AB Adult-Onset) OR (TI "insulin resistance" OR AB "insulin resistance"))

**#3:**

(MH "Self Care+") OR (MH "Health Behavior+") OR (MH "Assistive Technology Devices") OR (MH "disease management+") "OR" (MH "Exercise+") OR (MH "Technology") OR (MH "Life Style") OR (MH "Health Education+") OR (MH "Behavior Therapy+") OR (MH "Telemedicine+") OR (MH "Counseling+") OR (MH "Self Regulation+") OR  (MH “self efficacy") OR (MH "problem solving") OR (MH "decision making") OR (MH "Health Knowledge") OR (((TI problem OR AB problem) N3 (TI solving OR AB solving)) OR ((TI decision OR AB decision) N3 (TI making OR AB making))) OR (((TI self OR AB self) OR (TI symptom OR AB symptom) OR (TI medical OR AB medical) OR (TI disease OR AB disease) OR (TI emotional OR AB emotional)) N3 ((TI care OR AB care) OR (TI control OR AB control) OR (TI efficacy OR AB efficacy) OR (TI administ* OR AB administ*) OR (TI monitor* OR AB monitor*) OR (TI manage* OR AB manage*) OR (TI medicat* OR AB medicat*) OR (TI help OR AB help) OR (TI regulat* OR AB regulat*))) OR (((TI obes* OR AB obes*) OR (TI family OR AB family) OR (TI pain OR AB pain) OR (TI weight OR AB weight) OR (TI individual OR AB individual)) N3 ((TI manage* OR AB manage*) OR (TI program* OR AB program*) OR (TI reduc* OR AB reduc*))) OR ((TI cultur* OR AB cultur*) N3 ((TI appropriate OR AB appropriate) OR (TI tailor* OR AB tailor*) OR (TI relevan* OR AB relevan*) OR (TI adapt* OR AB adapt*))) OR ((TI exercis* OR AB exercis*) OR (TI "physical activit*" OR AB "physical activit*") OR (TI "life style" OR AB "life style") OR (TI lifestyle OR AB lifestyle) OR (TI "Diabetes Prevention Program" OR AB "Diabetes Prevention Program") OR (TI DSMP OR AB DSMP) OR (TI DPP OR AB DPP) OR (TI DSME* OR AB DSME*) OR (TI DSMS OR AB DSMS) OR (TI technol* OR AB technol*) OR (TI educat* OR AB educat*) OR (TI counsel* OR AB counsel*) OR (TI coach* OR AB coach*) OR (TI "health behavior*" OR AB "health behavior*") OR (TI therap* OR AB therap*) OR (TI technolog* OR AB technolog*) OR (TI telemedicine OR AB telemedicine) OR (TI telehealth OR AB telehealth))

**#4:**

(#1 AND #2 AND #3)

**EMBASE**

**Date searched:**  December 7, 2020
**Number of Results:** 617

**Date searched:** June 17, 2021
**Number of results:** 4
**Date filter:** 2021

**#1:**

'Asian American'/de OR ((asia* OR korea* OR chinese OR china OR vietnam* OR hmong OR filipino* OR indian* OR japan* OR burm* OR nepal* OR bangladesh* OR indonesia* OR pakistan* OR malaysia* OR myanmar OR cambod* OR singapor*) NEAR/3 american*):ti,ab,kw

**#2:**

'non insulin dependent diabetes mellitus'/de OR 'insulin resistance'/de OR diabet*,kw.:ti,ab OR (type2 OR T2D OR T2DM OR "type 2" OR type-2 OR "type II" OR type-II OR TIID OR NIDDM OR "NonInsulin Dependent" OR "Non-Insulin Dependent" OR Adult-Onset OR "insulin resistance"):ti,ab,kw

**#3:**

'Self Care'/exp OR 'Health Behavior'/exp OR 'Self Help Device'/exp OR 'disease management'/de OR 'Exercise'/exp OR 'Technology'/exp OR 'Lifestyle'/exp OR 'Health Education'/exp OR 'Behavior Therapy'/exp OR 'Telemedicine'/exp OR 'Counseling'/exp OR 'Self Control'/exp OR ‘self concept’/de OR 'problem solving'/de OR 'patient decision making'/exp OR ((problem NEAR/3 solving) OR (decision NEAR/3 making)):ti,ab,kw OR ((self OR symptom OR medical OR disease OR emotional) NEAR/3 (care OR control OR efficacy OR administ* OR monitor* OR manage* OR medicat* OR help OR regulat*)):ti,ab,kw OR ((obes* OR family OR pain OR weight OR individual) NEAR/3 (manage* OR program* OR reduc*)):ti,ab,kw OR (cultur* NEAR/3 (appropriate OR tailor* OR relevan* OR adapt*)):ti,ab,kw OR (exercis* OR "physical activit*" OR "life style" OR lifestyle OR "Diabetes Prevention Program" OR DSMP OR DPP OR DSME* OR DSMS OR technol* OR educat* OR counsel* OR coach* OR "health behavior*" OR therap* OR technolog* OR telemedicine OR telehealth):ti,ab,kw

**#4:**

(#1 AND #2 AND #3)

**PsycInfo**

**Date searched:**  December 7, 2020
**Number of Results:** 113

**Date searched:** June 18, 2021
**Number of results:** 2
**Date filter:** 2021

**#1:**

(DE "Asians") OR (((TI asia* OR AB asia*) OR (TI korea* OR AB korea*) OR (TI chinese OR AB chinese) OR (TI china OR AB china) OR (TI vietnam* OR AB vietnam*) OR (TI hmong OR AB hmong) OR (TI filipino* OR AB filipino*) OR (TI indian* OR AB indian*) OR (TI japan* OR AB japan*) OR (TI burm* OR AB burm*) OR (TI nepal* OR AB nepal*) OR (TI bangladesh* OR AB bangladesh*) OR (TI indonesia* OR AB indonesia*) OR (TI pakistan* OR AB pakistan*) OR (TI malaysia* OR AB malaysia*) OR (TI myanmar OR AB myanmar) OR (TI cambod* OR AB cambod*) OR (TI singapor* OR AB singapor*)) N3 (TI american* OR AB american*))

**#2:**

(DE "Type 2 Diabetes") OR (TI diabet*,kw. OR AB diabet*,kw.) OR ((TI type2 OR AB type2) OR (TI T2D OR AB T2D) OR (TI T2DM OR AB T2DM) OR (TI "type 2" OR AB "type 2") OR (TI type-2 OR AB type-2) OR (TI "type II" OR AB "type II") OR (TI type-II OR AB type-II) OR (TI TIID OR AB TIID) OR (TI NIDDM OR AB NIDDM) OR (TI "NonInsulin Dependent" OR AB "NonInsulin Dependent") OR (TI "Non-Insulin Dependent" OR AB "Non-Insulin Dependent") OR (TI Adult-Onset OR AB Adult-Onset) OR (TI "insulin resistance" OR AB "insulin resistance"))

**#3:**

(DE "Self-Care") OR (DE "Self-Care Skills”) OR DE "Health Behavior" OR DE "Health Risk Behavior" OR DE "Preventive Health Behavior" OR  (MH "disease management") "OR" (DE "Exercise") OR (DE "Technology") OR (DE "Lifestyle") OR (DE "Health Education") OR (DE "Behavior Therapy") OR (DE "Telemedicine+") OR (DE "Counseling") OR (MH "Self-Control") OR (DE "self-efficacy") OR (DE "problem solving") OR (DE "decision making") OR (DE "Health Knowledge”) OR (DE “Health Attitudes”) OR (((TI problem OR AB problem) N3 (TI solving OR AB solving)) OR ((TI decision OR AB decision) N3 (TI making OR AB making))) OR (((TI self OR AB self) OR (TI symptom OR AB symptom) OR (TI medical OR AB medical) OR (TI disease OR AB disease) OR (TI emotional OR AB emotional)) N3 ((TI care OR AB care) OR (TI control OR AB control) OR (TI efficacy OR AB efficacy) OR (TI administ* OR AB administ*) OR (TI monitor* OR AB monitor*) OR (TI manage* OR AB manage*) OR (TI medicat* OR AB medicat*) OR (TI help OR AB help) OR (TI regulat* OR AB regulat*))) OR (((TI obes* OR AB obes*) OR (TI family OR AB family) OR (TI pain OR AB pain) OR (TI weight OR AB weight) OR (TI individual OR AB individual)) N3 ((TI manage* OR AB manage*) OR (TI program* OR AB program*) OR (TI reduc* OR AB reduc*))) OR ((TI cultur* OR AB cultur*) N3 ((TI appropriate OR AB appropriate) OR (TI tailor* OR AB tailor*) OR (TI relevan* OR AB relevan*) OR (TI adapt* OR AB adapt*))) OR ((TI exercis* OR AB exercis*) OR (TI "physical activit*" OR AB "physical activit*") OR (TI "life style" OR AB "life style") OR (TI lifestyle OR AB lifestyle) OR (TI "Diabetes Prevention Program" OR AB "Diabetes Prevention Program") OR (TI DSMP OR AB DSMP) OR (TI DPP OR AB DPP) OR (TI DSME* OR AB DSME*) OR (TI DSMS OR AB DSMS) OR (TI technol* OR AB technol*) OR (TI educat* OR AB educat*) OR (TI counsel* OR AB counsel*) OR (TI coach* OR AB coach*) OR (TI "health behavior*" OR AB "health behavior*") OR (TI therap* OR AB therap*) OR (TI technolog* OR AB technolog*) OR (TI telemedicine OR AB telemedicine) OR (TI telehealth OR AB telehealth))

**#4:**

 (#1 AND #2 AND #3)
