# Supplementary material for: Type 2 Diabetes Self-Management Interventions Among Asian Americans in the United States: A Scoping Review
Source: Health Equity. 2022 Sep 23;6(1):750–66. doi: 10.1089/heq.2021.0083 (PMC9536350; doi:10.1089/heq.2021.0083)
Supplement: Supplemental data [file Suppl_AppSA2.docx]

**Supplemenatry Appendix 2:**

**Intervention and control description (if applicable), outcomes description and outcome values.**

| **Author** | **Asian American group** | **Intervention (I) arm** | |  | **Control (C) arm** | | **Primary, secondary, or other outcomes** | **Outcomes values**  **M(SD) or ±SE**  **I: Intervention C: Control** | |  |
| --- | --- | --- | --- | --- | --- | --- | --- | --- | --- | --- |
|  |  | N |  |  | N |  |  | Baseline | P2: 4-6 months | |
| Bender et al., 2017 | Filipino Americans | 22 | PilAm Go4Health (including Private Facebook group and follow-up maintenance) |  | 23 | Active Waitlist Control Group (received intervention in Phase 2). | Feasibility engagement and adherence to mHealth engagement measures | na | na | |
|  |  |  |  |  |  |  | HbA1C (mg/dL) | I: 7.4(0.82)  C: 7.4(0.93) | 7.1(0.98)  7.1(1.2) | |
|  |  |  |  |  |  |  | FBG (mg/dl) | I: 133(20.8)  C:137.4(30.1) | 128.7(30.6)  132.0(33.0) | |
|  |  |  |  |  |  |  | Weight (kg) | I: 72.6(10.8)  C:78.8(18.6) | 70.8(11.0)  76.4(19.8) | |
|  |  |  |  |  |  |  | BMI (kg/m2) | I: 28.5(3.6)  C:31.5(5.1) | 27.8(3.6)  30.5(5.6) | |
|  |  |  |  |  |  |  | Waist circumference (cm) | I: 97.1(8.7)  C:101.9(12.1) | 94.2(9.5)  99.9(13.0) | |
|  |  |  |  |  |  |  | Exercise (steps/day) | I: 7483 (2415)  C:6735(2363) | 9524 (3626)  7208(2719) | |
|  |  |  |  |  |  |  |  | Pre-treatment | T4: 32 weeks post-treatment | |
| Chesla et al., 2014 | Chinese Americans | 178 | Chinese Coping Skills Training (CCST) – DSME in Cantonese (examining gender differences) |  | N/A | N/A | Diabetes self-efficacy (higher scores means higher efficacy) | ♀:28.8(4.36)  ♂:28.6(4.57) | 29.2  30.1 | |
|  |  |  |  |  |  |  | Bicultural efficacy (higher score means higher efficacy) | ♀:29.6(4.37)  ♂:29.4(4.10) | 29.5  29.8 | |
|  |  |  |  |  |  |  | Family instrumental support (1-5; higher score means higher support) | ♀:3.9(0.90)  ♂:3.9(0.79) | 4.08  4.18 | |
|  |  |  |  |  |  |  | DQoL-Satisfaction (higher score means higher quality of life) | ♀:49.7(8.36)  ♂:50.6(7.43) | 51.0  53.0 | |
|  |  |  |  |  |  |  |  |  |  | |
|  |  |  |  |  |  |  |  | Pre-treatment | *T3-T4 (post-treatment)*  *(B, SE)* | |
| Chesla et al., 2013 | Chinese Americans | 145 | Chinese Coping Skills Training (CCST) - CBT |  | N/A | N/A | Acceptability and satisfaction | na | na | |
|  |  |  |  |  |  |  | Diabetes self-efficacy (higher score means higher efficacy) | 28.95(4.22) | 1.67(.33) | |
|  |  |  |  |  |  |  | Diabetes knowledge (%) | 0.54(0.24) | .07(.01) | |
|  |  |  |  |  |  |  | Bicultural efficacy (higher score means higher efficacy) | 29.58(4.11) | 1.05(.33) | |
|  |  |  |  |  |  |  | Family conflict resolution (higher score means more unresolved conflict) | 9.86(4.38) | -.28(.36) | |
|  |  |  |  |  |  |  | Family support–emotional (1-5; higher score means higher support) | 4.02(0.69) | .12(.05) | |
|  |  |  |  |  |  |  | Family support-instrumental (1-5; higher score means higher support) | 3.94(0.86) | .14(.07) | |
|  |  |  |  |  |  |  | Diabetes distress (higher means higher distress) | 2.59(2.38) | -.22(.08) | |
|  |  |  |  |  |  |  | DQoL-satisfaction (higher score means higher quality of life) | 50.01(7.52) | 1.78(.57) | |
|  |  |  |  |  |  |  | DQoL-impact (higher score means lower quality of life) | 41.85(7.84) | -90(.50) | |
|  |  |  |  |  |  |  | HbA1C (mg/dL) | 7.17(1.28) | .01(.01) | |
|  |  |  |  |  |  |  |  | Intervention  Pre | Intervention  Post | |
| Culhane- Pera et al., 2005 | Hmong Americans | 39 | Group visits |  | 22  216 | Refusers  Usual diabetes care | HbA1C (%, mean) | 9.46 | 9.58 | |
|  |  |  |  |  |  |  | TG (mg/dL) | 245.04 | 193.65 | |
|  |  |  |  |  |  |  | Cholesterol, total (mg/dL) | 214.86 | 200.54 | |
|  |  |  |  |  |  |  | HDL (mg/dL) | 43.11 | 41.07 | |
|  |  |  |  |  |  |  | LDL (mg/dL) | 127.86 | 125.33 | |
|  |  |  |  |  |  |  | BUN (mg/dL) | 21.89 | 20.81 | |
|  |  |  |  |  |  |  | Creatinine (mg/dL) | 1.39 | 1.32 | |
|  |  |  |  |  |  |  | Microalbumin/creatinine (mg/g) | 175.51 | 185.54 | |
|  |  |  |  |  |  |  | SBP (mmHg) | 132.67 | 127.56 | |
|  |  |  |  |  |  |  | DBP (mmHg) | 78.06 | 78.64 | |
|  |  |  |  |  |  |  | BMI (kg/m^2^) | 28.04 | 27.92 | |
|  |  |  |  |  |  |  | Depression/Anxiety scale (mean, 1.75 means impaired mental health) | 1.04 | 0.77 | |
|  |  |  |  |  |  |  |  |  |  |  |
|  |  |  |  |  |  |  |  |  |  | |
|  |  |  |  |  |  |  |  | Intervention  Baseline % or mean (95%CI) | Post-intervention  % or mean (95% CI) | |
| Ho et al., 2021 | Chinese Americans | 15 | Integrative Nutrition Counseling (INC) + Biomedical nutrition concepts |  | N/A | N/A | Acceptability | na | na | |
|  |  |  |  |  |  |  | Diabetes self-care: diet (mean number of days) | 5.00  (4.26,5.74) | 5.00  (4.13,5.87) | |
|  |  |  |  |  |  |  | Diabetes distress (95%CI; 1-6:lower score is more optimal) | 2.89  (2.06, 3.72) | 2.94  (2.21,3.67) | |
|  |  |  |  |  |  |  | Diabetes self-efficacy (95%CI; higher score is more optimal) | 76.89  (64.14, 89.64) | 80.00  (72.47,87.53) | |
|  |  |  |  |  |  |  | HbA1c | Described qualitatively | Described qualitatively | |
|  |  |  |  |  |  |  |  |  |  |  |
|  |  |  |  |  |  |  |  |  |  |  |
|  |  |  |  |  |  |  |  | Baseline,  M ± SE | Mean change at 6 months ± SE |  |
| Ho et al., 2020 | Chinese Americans | 11 | Diabetes education with INC |  | 7 | Usual diabetes self-management education (DSME) | Feasibility | na | na |  |
|  |  |  |  |  |  |  | Diabetes self-efficacy (higher score indicate more optimal outcome) | I: 31.1±1.5  C:33.0±1.8 | 1.1±1.4  0.8±1.8 |  |
|  |  |  |  |  |  |  | Bicultural self-efficacy (higher score indicate more optimal outcome) | I: 32.2±1.6  C:32.9±1.5 | 1.2±1.5  0.6±1.9 |  |
|  |  |  |  |  |  |  | Eating self-efficacy (single item) (higher score indicate more optimal outcome) | I: 3.8±0.3  C:3.1±0.4 | 0.3±0.5  0.5±0.6 |  |
|  |  |  |  |  |  |  | Family support-instrumental (higher score indicate more optimal outcome) | I: 4.3±0.2  C:4.0±0.3 | –0.01±0.2  0.3±0.3 |  |
|  |  |  |  |  |  |  | Family support – emotional (higher score indicate more optimal outcome) | I: 3.2±0.2  C:3.6±0.2 | –0.2±0.3  –0.1±0.3 |  |
|  |  |  |  |  |  |  | DQoL – satisfaction (higher score indicate more optimal outcome) | I: 55.8±2.8  C:61.4±3.5 | –1.8±2.2  1.0±2.7 |  |
|  |  |  |  |  |  |  | Diabetes distress (lower score indicate more optimal outcome) | I: 1.8±0.3  C:2.1±0.3 | –0.02±0.3  –0.02±0.3 |  |
|  |  |  |  |  |  |  | Dietary adherence (Mediterranean diet; (higher score indicate more optimal outcome) | I: 55.6±2.6  C:54.3±3.3 | 4.3±1.4  –5.5±1.8 |  |
|  |  |  |  |  |  |  | HbA1C (mg/dL) | I: 7.0±0.3  C:7.5±0.4 | –0.4+0.3  –0.6+0.3 |  |
|  |  |  |  |  |  |  | Weight, lbs | I: 135.7±8.3  C:130.0±10.3 | –0.5+0.8  0.1±1.0 |  |
|  |  |  |  |  |  |  |  | PreSession  *mean ± SE* | EndSession (12 months)  *mean ± SE* |  |
| Inouye et al., 2012 | Asian Americans | 77 | Cognitive Behavioral Therapy (CBT) |  | 90 | Diabetes Education and Support (DES) | HbA1C (%) | I: 7.93  C:7.79 | 7.84  7.79 |  |
|  |  |  |  |  |  |  | TG (mg/dL) | I: 162.7  C:175.2 | 163.7  177.2 |  |
|  |  |  |  |  |  |  | Cholesterol, total (mg/dL) | I: 165.5  C:167.6 | 174.1  172.8 |  |
|  |  |  |  |  |  |  | HDL (mg/dL) | I: 44.4  C: 42.5 | 43.5  45.8 |  |
|  |  |  |  |  |  |  | LDL (mg/dL) | I: 89.3  C: 93.3 | 86.9  87.8 |  |
|  |  |  |  |  |  |  | SBP (mmHg) | I: 136.0  C:136.5 | 135.4  136.2 |  |
|  |  |  |  |  |  |  | DBP (mmHg) | I: 84.1  C:80.1 | 81.3  81.4 |  |
|  |  |  |  |  |  |  | Weight, lbs | I: 185.6  C:188.8 | 191.8  191.2 |  |
|  |  |  |  |  |  |  | BMI (kg/M^2^) | I: 31.3  C:31.4 | 32.0  31.7 |  |
|  |  |  |  |  |  |  | Self-efficacy (0-100; higher value indicates better results) | I: 58.47±2.30  C:65.26±2.33 | 62.60±2.61  66.06±244 |  |
|  |  |  |  |  |  |  | Health belief (cues to action)^e^; higher indication more non-compliant) | I: 12.12±0.29  C:12.26±0.29 | 12.08±0.32  12.60±0.30 |  |
|  |  |  |  |  |  |  | Depression (CESD) (A score of >16 suggest a clinically significant level of psychological distress) | I: 10.48±0.83  C:9.68±0.83 | 9.33±0.90  10.21±0.86 |  |
|  |  |  |  |  |  |  | DQoL-satisfaction (0-100; higher score indicates better quality of life) | I: 61.10±1.66  C:62.46±1.65 | 62.77±1.85  65.20±1.72 |  |
|  |  |  |  |  |  |  | DQoL – impact (0-100; higher score indicates better quality of life) | I: 76.49±1.26  C: 72.96±1.25 | 77.23±1.38  76.24±1.30 |  |
|  |  |  |  |  |  |  | General Health (0-100; higher score indicates favorable health state) | I: 54.34±2.07  C: 52.96±2.05 | 57.80±2.23  58.61±2.11 |  |
|  |  |  |  |  |  |  |  | Baseline  N(%) | 12 months  N (%) |  |
| Islam et al., 2013 | Bangladeshi Americans | 26 | CHW-facilitated group session (self-help intervention) |  | N/A | N/A | General health (higher score = excellent) | 11(42.3) | 22(84.6) |  |
|  |  |  |  |  |  |  | Physical activity (Several days/everyday) | 13(52.0) | 23(88.5) |  |
|  |  |  |  |  |  |  | Diet (higher score = very confident to stay healthy diet) | 16(61.5) | 18(69.2) |  |
|  |  |  |  |  |  |  |  | Baseline  M(SD) | 12 months  M(SD) |  |
|  |  |  |  |  |  |  | HbA1C (mg/dL) | 7.6(1.3) | 7.1(0.8) |  |
|  |  |  |  |  |  |  | BMI (kg/M^2^) | 29.1(6.8) | 28.6(6.6) |  |
|  |  |  |  |  |  |  | Weight, lbs | 157.4(30.8) | 154.8(30.1) |  |
|  |  |  |  |  |  |  | Mental health(PHQ-2) (0=Low Risk, 6 = High Risk) | 2.6(1.9) | 0.9(1.6) |  |
|  |  |  |  |  |  |  | Diabetes knowledge (13 highest score) | 7.4(2.0) | 10.9(1.3) |  |
|  |  |  |  |  |  |  | Self-efficacy in health access (1=Low, 5=High) | 4.0(0.9) | 4.4(0.7) |  |
|  |  |  |  |  |  |  | Medication compliance (8=perfect compliance) | 11.2±3.6 | 9.5±2.0 |  |
|  |  |  |  |  |  |  |  | Baseline  M(SD) | 6 months  M(SD) |  |
| Ivey et al., 2012^a^ | Chinese Americans | 46 | Ethnic and language-concordant teamlet model by using health coaches tailored for Chinese patients |  | 46 | Usual care | HbA1C (mg/dL) | I: 7.60(1.16)  C:7.62(0.75) | 7.24(1.02)  7.63(1.23) |  |
|  |  |  |  |  |  |  |  | Baseline (SE) | 12 months  (SE) |  |
| Kim et al., 2016 | Korean Americans | 120 | Self-help intervention program – diabetes mellitus (SHIP-DM) delivered by RN or Community Health Worker (CHW) |  | 130 | Usual | HbA1C (mg/dL) | CHW: 9.1(0.2) RN: 8.6(0.2)  Usual: 8.8(0.1) | 7.5(0.2)  7.6(0.2)  8.1(0.1) |  |
|  |  |  |  |  |  |  | Blood glucose level (mg/dL) | CHW: 175.1(6.9)  RN: 144.1(7.1)  Usual:158.3(5.0) | 125.1(6.9)  124.6(7.1)  147.1(5.0) |  |
|  |  |  |  |  |  |  | Diabetes self-efficacy (higher score indicate better results) | CHW: 48.8(1.9)  RN: 49.5(2.0)  Usual:44.7(1.4) | 58.5(1.9)  58.7(2.0)  46.5(1.4) |  |
|  |  |  |  |  |  |  | DQoL (range 0-75; higher score indicates better quality of life) | CHW: 48.5(1.3)  RN: 51.8(1.4)  Usual:51.0(1.0) | 57.1(1.3)  58.1(1.4)  49.9(1.0) |  |
|  |  |  |  |  |  |  |  | Baseline  M(SE) | 12 months  M(SE) |  |
| Kim et al., 2015 | Korean Americans | 105 | Community-based multi-modal behavioral self-help intervention program (SHIP-DM) |  | 104 | Delayed intervention after month12 | HbA1C (%) | I: 8.9(0.2)  C:8.8(0.2) | 7.6(0.1)  8.1(0.1) |  |
|  |  |  |  |  |  |  | Fasting Blood Glucose (mg/dL) | I: 160.1(6.3)  C:158.3(5.3) | 124.8(4.3)  147.1(4.9) |  |
|  |  |  |  |  |  |  | TG (mg/dL) | I: 177.3(11.2)  C:180.6(12.6) | 167.6(10.3)  174.7(12.3) |  |
|  |  |  |  |  |  |  | Cholesterol, Total (mg/dL) | I: 188.5(4.2)  C:201.1(5.1) | 178.8(3.7)  193.6(4.7) |  |
|  |  |  |  |  |  |  | HDL (mg/dL) | I: 49.8(1.2)  C:48.7(1.1) | 49.0(1.2)  49.7(1.2) |  |
|  |  |  |  |  |  |  | LDL (mg/dL) | I: 104.6(3.3)  C:115.6(4.0) | 97.6(3.1)  109.1(3.5) |  |
|  |  |  |  |  |  |  | SBP (mmHg) | I: 134.7(1.8)  C:133.1(2.0) | 134.3(1.5)  136.2(1.9) |  |
|  |  |  |  |  |  |  | DBP (mmHg) | I: 78.9(1.0)  C:78.5(1.1) | 79.0(0.9)  81.2(0.9) |  |
|  |  |  |  |  |  |  | Diabetes self-efficacy (range: 0-80; higher score = better results) | I: 49.1(1.3)  C:44.7(1.5) | 58.6(1.2)  46.5(1.6) |  |
|  |  |  |  |  |  |  | Diabetes knowledge  (range: 0-14; higher score = better results) | I: 7.6(0.3)  C:7.8(0.3) | 10.3(0.2)  8.3(0.3) |  |
|  |  |  |  |  |  |  | Quality of life  (range: 0-75; higher score = better quality of life) | I: 50.1(1.1)  C:51.0(1.1) | 57.6(1.0)  49.9(1.0) |  |
|  |  |  |  |  |  |  | Attitudes toward diabetes (10-50; higher score = improvement) | I: 32.7(0.7)  C:34.4(0.7) | 34.7(0.8)  32.8(0.7) |  |
|  |  |  |  |  |  |  | Depression  (range: 0-27; lower score = more optimal outcome) | I: 5.3(0.5)  C:5.4(0.5) | 4.8(0.5)  4.1(0.4) |  |
|  |  |  |  |  |  |  |  | Baseline  M(SD) | Change from baseline 30 Weeks |  |
| Kim et al., 2009 | Korean Americans | 41 | Community-based, culturally tailored behavioral intervention (SHIP-DM) |  | 42 | Delayed intervention | HbA1C (mg/dL) | I: 9.4(1.5)  C:9.1(1.3) | –1.3(1.3)  –0.4(1.4) |  |
|  |  |  |  |  |  |  | Fasting blood glucose (mg/dL) | I: 188.3(56.8)  C:169.3(61.6) | –42.3(61.6)  –7.0(57.2) |  |
|  |  |  |  |  |  |  | TG(mg/dL) | I: 246.6(366.8)  C:160.9(108.0) | –84.6(384.4)  –4.2(115.8) |  |
|  |  |  |  |  |  |  | Cholesterol (mg/dL) | I: 207.0(36.3)  C:179.8(36.6) | –24.7(41.9)  7.2(37.2) |  |
|  |  |  |  |  |  |  | HDL (mg/dL) | I: 47.6(10.1)  C:49.7(13.7) | –2.5(6.5)  0.6(10.3) |  |
|  |  |  |  |  |  |  | LDL(mg/dL) | I: 124.1(37.7)  C:71.8(181.2) | –15.9(38.7)  35.6(185.7) |  |
|  |  |  |  |  |  |  | SBP (mmHg) | I: 131.5(14.1)  C:132.7(17.2) | –0.2(19.7)  –3.6(16.6) |  |
|  |  |  |  |  |  |  | DBP (mmHg) | I: 80.8(8.8)  C:77.9(9.1) | –0.3(12.3)  0.7(10.8) |  |
|  |  |  |  |  |  |  | BMI (kg/m^2^) | I: 25.9(3.1)  C:25.7(3.4) | –0.3(1.2)  –0.3(1.2) |  |
|  |  |  |  |  |  |  | Diabetes knowledge (higher score = more optimal outcome) | I: 7.7(2.8)  C:7.8(3.1) | 2.4(2.3)  0.3(3.7) |  |
|  |  |  |  |  |  |  | Self-efficacy (higher score = higher levels of self-efficacy) | I: 48.5(12.2)  C:47.3(13.6) | 6.6(14.4)  –0.9(15.1) |  |
|  |  |  |  |  |  |  | Self-care activities (higher score = more optimal outcome) | I: 50.6(15.9)  C:541(16.1) | 17.5(16.9)  2.5(15.4) |  |
|  |  |  |  |  |  |  | Depression (higher score indicates higher frequency of depression symptoms) | I: 4.8(4.4)  C:6.4(5.8) | –0.5(4.5)  –1.0(4.3) |  |
|  |  |  |  |  |  |  | DQoL (lower score indicate more satisfaction) | I: 88.6(19.2)  C:96.5(24.1) | –4.6(17.3)  0.3(16.4) |  |
|  |  |  |  |  |  |  | Attitudes toward diabetes (lower score = more optimal outcome) | I: 25.9(7.0)  C:28.1(8.7) | –3.7(7.1)  –1.4(6.5) |  |
|  |  |  |  |  |  |  |  |  |  |  |
| Kwan et al., 2014 | Chinese Americans | 163 | Culturally adapted diabetes intervention |  | N/A | N/A | HbA1C | Reported decrease in value (per narrative results) |  |  |
|  |  |  |  |  |  |  |  | Baseline  M(SD) | 12 months |  |
| Le et al., 2013 | Chinse Americans | 109 | Linguistic and cultural knowledge of staff with culturally appropriate diabetes management program (Asian clinic) |  | 218 | Adult Diabetes Clinic | HbA1C (mg/dL) | I: 8.2(1.9)  C:7.9(1.8) | 7.4(1.3)  7.9(1.8) |  |
|  |  |  |  |  |  |  | MD/NP visits | I: Not reported  C: | 2.7(1.0)  3.1(1.5) |  |
|  |  |  |  |  |  |  | Missed MD/NP visits | I: Not reported  C: | 0.1(0.3)  0.1(0.3) |  |
|  |  |  |  |  |  |  | Education visits | I: Not reported  C: | 2.8(2.0)  3.3(3.3) |  |
|  |  |  |  |  |  |  | Missed education visits | I: Not reported  C: | 0.1(0.3)  0.3(0.6) |  |
|  |  |  |  |  |  |  |  |  |  |  |
|  |  |  |  |  |  |  |  |  |  |  |
|  |  |  |  |  |  |  |  | Baseline M(SD) | 30 weeks follow-up |  |
| Song et al., 2010 | Korean Americans | 40 | Culturally-tailored Nutritional education program (self-help intervention – SHIP-DM) |  | 39 | Delayed intervention | Nutrition/diabetes knowledge (higher score = increase knowledge) | I: 3.1(1.9)  C:3.1(1.8) | 4.4(1.9)  3.2(1.8) |  |
|  |  |  |  |  |  |  |  | Baseline  M(SD) | 6 months |  |
| Tomioka et al., 2014^b^ | Filipino Americans | 82 | DSMP (community clinic-based program) |  | N/A | N/A | HbA1C (%) | 6.9(0.70) | 6.3(0.51) |  |
|  |  |  |  |  |  |  | Fasting blood glucose (mg/dL) | 138.80(30.11) | 116.98(27.56) |  |
|  |  |  |  |  |  |  | TG (mg/dL) | 139.06(46.96) | 125.31(40.18) |  |
|  |  |  |  |  |  |  | Cholesterol (mg/dL) | 195.72(34.88) | 171.41(35.31) |  |
|  |  |  |  |  |  |  | HDL (mg/dL) | 58.84(14.37) | 52.23(13.89) |  |
|  |  |  |  |  |  |  | LDL (mg/dL) | 105.21(27.21) | 92.14(28.46) |  |
|  |  |  |  |  |  |  | SBP (mmHg) | 134.32(13.77) | 124.43(11.09) |  |
|  |  |  |  |  |  |  | DBP (mmHg) | 76.89(8.58) | 72.42(7.43) |  |
|  |  |  |  |  |  |  | BMI (kg/m^2^) | 25.20(3.41) | 24.43(3.06) |  |
|  |  |  |  |  |  |  | Exercise (aerobic; minutes spent in exercise) | 105.18(65.85) | 149.09(89.17) |  |
|  |  |  |  |  |  |  | Glucose monitoring (days/week) | 0.82(1.84) | 2.61(2.28) |  |
|  |  |  |  |  |  |  | Diabetes self-efficacy (0 = not at all confident, 10 = totally confident) | 6.89(0.90) | 9.46(0.40) |  |
|  |  |  |  |  |  |  | Coping skills (0 = never proactive; 5= always proactive) | 1.38(0.89) | 2.46(0.79) |  |
|  |  |  |  |  |  |  | Health distress (0= none of the time; 5 = all of the time) | 2.30(1.39) | 1.08(0.73) |  |
|  |  |  |  |  |  |  | Self-rated health (0=poor; 5 = excellent) | 3.12(0.79) | 1.87(0.58) |  |
|  |  |  |  |  |  |  |  | Baseline  M(SD) | 3 months  M(SD) |  |
| Wang et al., 2005^c^ | Chinese Americans | 33 | Culturally-tailored Diabetes Management Intervention program (dietary education, exercise, self-care, medication |  | N/A | N/A | HbA1C (mg/dL) | 7.11(1.1) | 6.12(2.4) |  |
|  |  |  |  |  |  |  | Weight (lbs) | 139.3(26.7) | 121.8(48.6) |  |
|  |  |  |  |  |  |  | SBP (mmHg) | 131.5(13.6) | 113.7(46.2) |  |
|  |  |  |  |  |  |  | DBP (mmHg) | 69.4(10.9) | 63.2(25.9) |  |
|  |  |  |  |  |  |  |  |  |  |  |
|  |  |  |  |  |  |  |  | Baseline CBT  M±SE or (SD) | 1 year CBT  M±SE |  |
| Yomogida et al., 2015^d^ | Asian Americans | 104 | Cognitive Behavioral intervention |  | 103 | Usual care | Caloric intake (Total kcals) | 1681 | Not reported |  |
|  |  |  |  |  |  |  | Average daily consumption of trans-fat (kcals) | 7.57±7.69 | 1.15±9.27 |  |
|  |  |  |  |  |  |  | Average daily consumption of sugar (kcals) | 58.13±7.48 | 48.63±8.50 |  |
|  |  |  |  |  |  |  | Exercise (number of steps) | 4721(2812) | Not reported |  |

^a^ shows the values for the larger sample size (*n*=92).

^b^ Other outcomes reported included level of symptoms, diabetes related symptoms, activities limitations (mean difference 0.91(1.13), p < .001), self-rated physical activities (mean difference 0.38(0.42), p < .001), communication with providers, mean difference -.04(0.87), NS) and healthcare utilization.

^c^ Study also reported that participants demonstrated healthy food choices (82.%), benefits of frequent exercise (90%), ways to prevent complications (82.5%), demonstrate stress management skills (70%), verbalize plan for skin and foot care (90%), self-management on sick days or travel (70%), accurate self-monitoring blood glucose skills (57.5%) and understanding of oral hypoglycemic agents (55%).

^d^ trans-fat and sugar consumption results show change from baseline to 1 year for treatment group; exercise result shows the differences in number of steps for the treatment group at 1 year. Study shows other outcomes not shown in the table (e.g., alcohol, caffeine, diet fiber, saturated fat intake).

^e^  health belief reported using Heath Belief Scale. Only Cues to Action reported in this table.

***Abbreviations:***

♀: Female

♂: Male

BMI: Body Mass Index

C: Control

CBI: Cognitive behavioral intervention

CBT: Cognitive behavioral therapy

CCST: Chinese-adapted Coping Skills Training

CHW: Community healthcare worker-counseled group

CI: Confidence Interval

DES: Diabetes Education and Support

DQOL: Diabetes Quality of Life

DSME: Diabetes Self-Management Education

DSMP: Diabetes Self-Management Program

HbA1C: Hemoglobin A1C

HDL: High density lipoprotein

I: Intervention

INC: Integrated nutrition counseling

LDL: Low density lipoprotein

MD: Medical doctor

NA: Not applicable or not measured

NS: Statistically non-significant

P1: Phase 1 (baseline to 3 months)

P2: Phase 2 (4 to 6 months)

Pre: Pretreatment (T1-T3)

RN: Registered nurse

SBP: Systolic Blood Pressure

DBP: Diastolic Blood Pressure

T2D: Type 2 diabetes

T3-T4: Data measured at 16 weeks-24 weeks (Post-treatment)

T4-T5: Data measured at 24 weeks-32 weeks (Maintenance)

TG: Triglycerides
