# Supplementary material for: Type 2 Diabetes Self-Management Interventions Among Asian Americans in the United States: A Scoping Review
Source: Health Equity. 2022 Sep 23;6(1):750–66. doi: 10.1089/heq.2021.0083 (PMC9536350; doi:10.1089/heq.2021.0083)
Supplement: Supplemental data [file Suppl_AppSA3.docx]

**Supplementary Appendix 3: Intervention components and duration**

| **Author** | **Asian American group representation** | **Components** | **Coded components (based on ADA Standard 6 and 7 DSME)†** | **Duration** |
| --- | --- | --- | --- | --- |
| Bender et al., 2017 | Filipino Americans | Fitbit accelerometer (lifestyle balance and social networking, physical activity and healthy eating plan, physical exam, blood draw, app and diary training for tracking steps, food/calories, weight, Facebook group training)'; 1 month - progress report and coaching on healthy behaviors, physical active benefits, social support for physical activity, Filipino dancing, basketball, walking, monitoring physical activity steps; 2-month visit - coaching on healthy behaviors, benefits and ways of healthy eating and limiting fat, social support for healthy eating, food alternatives, monitoring weight; 3 month - relapse prevention, problem-solving, staying motivated, handling barriers to healthy behaviors | A, D, E, F, H, I, J | 12 weekly discussions for private Facebook group, phase 1 and phase 2 nondescriptive |
|  |  | Private Facebook group: weekly discussions (exercise, eating, weight, barriers to weight loss, healthy lifestyle behaviors, social support, medication adherence) |  |  |
|  |  | Tailored lifestyle education, feedback, coaching, and support during research visits |  |  |
|  |  | App - diary and self-report daily food/calorie intake and weekly weight |  |  |
| Chesla et al., 2014 | Chinese Americans | 6 sessions to learn and practice basics of T2DM disease management and coping skills to manage disease in complex social contexts | A, H | 6-week treatment provider after 16 weeks |
|  |  | Review of diabetes disease and management in Cantonese, community-based Chinese myths about diabetes, problem-solving, communication, conflict resolution, stress management |  |  |
| Chesla et al., 2013 | Chinese Americans | Introduction to CCST, group introductions, relaxation techniques, review of type 2 diabetes and management strategies, discuss common Chinese cultural myths about diabetes, problem solving skills, communication skills, conflict management skills, stress management: cognitive restructuring skills | A, H, I | 4 months |
|  |  | Diabetes Intervention (CCST sessions) |  | 6 weeks |
| Culhane-Pera et al., 2005 | Hmong Americans | Check in (30 mins)- register, blood and urine testing; group discussion (90 mins)- initial: ideas, attitudes, and behaviors, subsequent- mental health, causation, diet, exercises, medicines, final- evaluation of group visits; 1:1 station (60 mins): family physician- documented medical care, diabetes nurse educator- checked glucometers and reinforced discussion topic, nursing assistant- obtained vital signs, gave flu shots, arranged referrals; group exercise (30 mins) | A, C, D, K, H, J, K (referrals) | 3 monthly meetings, then quarterly (total of 7 visits over 13 months) |
|  |  | Culturally familiar group visit |  |  |
| Ho et al., 2021 | Chinese Americans | Diagnostic interview with acupuncturist, 2-h small group course taught in Chinese by bilingual diabetes educator/registered dietitian based on INC curriculum | A, F | 2 hours small group course |
| Ho et al., 2020 | Chinese Americans | DSME (1st class: with acupuncturist - assessed patient using traditional Chinese medicine, signs/symptoms; basics, definitions of diabetes, and exercise; 2nd class: by acupuncturist an RN diabetes educator - INC assignment: diabetes diet mainly focusing on identifying and counting carbohydrates and introducing the glucometer; 3rd class: learned about diabetes mediations and created individual-self-management plans). | A, B, C, D, F, I, J | 2 hours, 1x/weekly |
|  |  | Guidebook – 44-page color guide in English/Chinese (biomedical nutritional standards and culturally specific practical tips). |  |  |
| Inouye et al., 2012 | Asian Americans | CBT focused on stress management, biofeedback with relaxation, mood management, cognitive restructuring, empowerment, values clarification, decision making | A, H, I | 6 sessions |
| Islam et al., 2013 | Bangladeshi Americans | CHW-facilitated group sessions (1st: overview of diabetes, including myths and facts, disease-specific information, BG levels; subsequent sessions - topics in nutrition, physical activity, diabetes complications, stress, and family, social support, access to health care. Sessions in clinical and community settings and separated by gender). Reminder calendar was developed. | A, C, D, E, F, H, I, J | 6 monthly, 2.5 hour |
|  |  | One-on-one visits from CHW |  | 3 visits, 3, 5, 9 months for 90 minutes |
| Ivey et al., 2012 | Chinese Americans | Pre-visit: health coach routine assessment, data gathering, medication reconciliation, determination of patient agenda. Face-to-face education visit after MD visit. Teamlet huddle: MD communicates care plan to health coach. | A, B, F, H, I, J | 30-60 minutes for 2 weeks |
|  |  | Post-visit: health coach: reinforce key elements (nutrition, medication, diabetes), action plan for lifestyle modification and adherence, schedule follow-up, navigate referral system. |  | 2 weeks |
|  |  | Follow-up phone call by health coaches |  | 3 follow-ups |
|  |  | Dietitian (diabetes education visit) |  | 3 visits |
| Kim et al., 2016 | Korean Americans | Reported somewhere else | Reported somewhere else | 2-hr/weekly for 6 weeks |
| Kim et al., 2015 | Korean Americans | Series of structured behavioral education using group education format, ongoing self-monitoring of blood glucose (patient's knowledge of diabetes and its treatment, reduce risk factors, coping/enabling capacities through problem-solving, cognitive reframing, belief in self), psychosocial education, health literacy enhancing component (reading food labels, understand medical terminology, follow instructions to access resources); and individualized counseling using motivational interviewing (individualized treatment goal); self-monitoring of glucose, take measurements | A, C, F, G, H, I | Weekly 2-hr sessions over 6 weeks |
|  |  | Bilingual nurses/CHW provided motivational interviewing, RN- counseling. |  |  |
| Kim et al., 2009 | Korean Americans | 3 concurrent intervention components: 2-hour weekly education sessions for 6 weeks, home glucose monitoring with tele-transmission, and monthly telephone counseling by a bilingual nurse for 24 weeks (10-25 mins); 6 topics: (1) overview of type 2 diabetes and general diabetes management guidelines, (2) short- and long-term complications of uncontrolled type 2 diabetes, (3) healthy eating and nutrition, (4) reading food labels and exercise, (5) medications and food-drug interactions, and (6) problem solving and communication skills with a primary care physician. | A, B, C, D, E, F, H, I | 30 weeks |
| Kwan et al., 2014 | Chinese Americans | Diabetes intervention but components not reported | Individual components not reported | 4-month delayed treatment, data intake @ baseline, 8 weeks, 16 weeks. Post-intervention data collected @ 17 weeks and 24 weeks. |
| Le et al., 2013 | Chinse Americans | Visits - integrating unique physiology, cultural beliefs, and explanatory models of predominant Asian cultures | A, I | 12 months |
| Song et al., 2010 | Korean Americans | Two 2-hour nutrition education sessions, 1 week apart. 6-week, structured, in-class self-help education program. (T2D overview; T2D medication therapy; T2D-related complications and long-term  management; nutrition education for T2D control (two parts - individually tailored serving table, daily food diary, culture-specific food model); stress management and mental health for people with T2D) | A, B, E, F, H | 6 weeks |
| Tomioka et al., 2014 | Filipino Americans | Pre-workshop orientation by program leaders and a physician, Reinforcement of key messages in native languages, Graduation ceremony with family members invited, six-month reunion, DSMP components | A, J, K (graduation) | Weekly session, 1x reunion |
| Wang et al., 2005 | Chinese Americans | Dietary Education, Self-monitoring blood glucose, Exercise, Medication, preventing/treating diabetic complications, develop plans for sick days/traveling, skills of stress management Self-care (including foot care) | A, B, C, D, E, F, G, H, I, J | 10 weeks (10 educational sessions, once a week, 60 mins each) |
| Yomogida et al., 2015 | Asian Americans | Cognitive behavioral methods: 1. welcome, intro, orientation, self-management, personal setting of goals, baseline test of self-management knowledge; 2. cognitive restructuring, affect management, and management of cognitive distortions; 3. stress theory and management; 4. stress and relaxation; 5. cultural influences on behaviors; 6. time management, exit interview, post-treatment, questionnaire | A, C, D, F, H, I | 6-week CBI |

***Note:***

**†** Legend used under coded components: ***A:*** *Assess cultural beliefs, health beliefs, current knowledge, physical limitations, family support, financial status, medical history, literacy, numeracy, education, and knowledge; B: Medication, C: Monitoring blood glucose; D: Physical activity; E: Preventing, detecting, and treating acute and chronic complications; F: Nutrition; G: Risk reduction – e.g., smoking cessation, foot care; H – Developing personal strategies to promote health and behavior change; I: Developing personal strategies to promote health and behavior change; J: Review and reinforce treatment goals and self-management needs; K: Other*

***Abbreviations:***

CBI: Cognitive behavioral intervention

CBT: Cognitive behavioral therapy

CCST: Chinese-adapted Coping Skills Training

CHW: Community healthcare worker

DSME: Diabetes Self-Management Education

DSMP: Diabetes Self-Management Program

INC: Integrated nutrition counseling

MD: Medical doctor

RN: Registered nurse

T2D: Type 2 diabetes
